# What Can Causal Networks Tell Us about Metabolic Pathways?

**DOI:** 10.1371/journal.pcbi.1002458

**Published:** 2012-04-05

**Authors:** Rachael Hageman Blair, Daniel J. Kliebenstein, Gary A. Churchill

**Affiliations:** 1 State University of New York at Buffalo, Buffalo, New York, United States of America; 2 University of California, Davis, California, United States of America; 3 The Jackson Laboratory, Bar Harbor, Maine, United States of America; Cornell University, United States of America

## Abstract

Graphical models describe the linear correlation structure of data and have been used to establish causal relationships among phenotypes in genetic mapping populations. Data are typically collected at a single point in time. Biological processes on the other hand are often non-linear and display time varying dynamics. The extent to which graphical models can recapitulate the architecture of an underlying biological processes is not well understood. We consider metabolic networks with known stoichiometry to address the fundamental question: *“What can causal networks tell us about metabolic pathways?”*. Using data from an *Arabidopsis* Bay
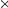
Sha population and simulated data from dynamic models of pathway motifs, we assess our ability to reconstruct metabolic pathways using graphical models. Our results highlight the necessity of non-genetic residual biological variation for reliable inference. Recovery of the ordering within a pathway is possible, but should not be expected. Causal inference is sensitive to subtle patterns in the correlation structure that may be driven by a variety of factors, which may not emphasize the substrate-product relationship. We illustrate the effects of metabolic pathway architecture, epistasis and stochastic variation on correlation structure and graphical model-derived networks. We conclude that graphical models should be interpreted cautiously, especially if the implied causal relationships are to be used in the design of intervention strategies.

## Author Summary

High-throughput profiling data are pervasive in modern genetic studies. The large-scale nature of the data can make interpretation challenging. Methods that estimate networks or graphs have become popular tools for proposing causal relationships among traits. However, it is not obvious that these methods are able to capture causal biological mechanisms. Here we address the power and limitations of causal inference methods in biological systems. We examine metabolic data from simulation and from a well-characterized metabolic pathway in plants. We show that variation has to propagate through the pathway for reliable network inference. While it is possible for causal inference methods to recover the ordering of the biological pathway, it should not be expected. Causal relationships create subtle patterns in correlation, which may be dominated by other biological factors that do not reflect the ordering of the underlying pathway. Our results shape expectations about these methods and explain some of the successes and failures of causal graphical models for network inference.

## Introduction

Understanding the nature of cause and effect is fundamental to all fields of scientific investigation, but the concept of causality can present special difficulties in biology [1]. Experiments that utilize controlled interventions represent the most widely used approach to establishing causality. However, in his seminal work on experimental design, RA Fisher proposed that causation can be inferred from multi-factorial experiments performed with randomization [2]. An extension of this principle provides the foundation for computational approaches to network reconstruction in experimental genetic crosses, such as the recombinant inbred strain panel used in this study. Natural allelic variation is randomized during meiosis to generate a multi-factorial perturbation affecting multiple phenotypic outcomes. This meiotic randomization allows for the inference of quantitative trait loci (QTL) that are causal to phenotype [3].

Recent advances in high-throughput phenotyping technologies have made large-scale measurements of molecular traits possible. Expression QTL (eQTL), metabolic QTL (mQTL) and protein QTL (pQTL) can be used to link thousands of molecular phenotypes to genetic loci, as well as to clinical phenotypes [4]. A typical xQTL study will involve cross sectional sampling of a genetically variable population at a single time point. It is not immediately obvious that such data could provide insight into causal biological mechanisms, which derive from non-linear dynamic processes of gene expression and metabolism. However, a rich body of literature supports the idea that correlation structure in static data can provide insights into causal relationships among the measured variables [5], [6].

The interpretation of a directed edge between nodes 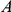 and 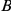 in a graphical model is that intervention on 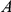 will alter 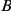, but intervention on 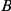 will not alter 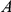. In a metabolic reaction, intervention on the substrate concentration will alter the product concentration. Reaction stoichiometry is often well understood [7]. Substrate molecules are converted by known enzymes into products, which in turn act as substrates for subsequent reactions. Reactions are organized into pathways which may converge, branch or intersect to form elaborate networks. More complex pathways involving feedback through allosteric interactions between enzymes and metabolites may also be present. It is not clear to what extent graphical models inferred from mQTL data capture these types of interactions.

Several algorithms have been proposed for the inference of causal relationships among phenotypes using genetic data [8]–[14]. These methods employ linear statistical models to infer the relationships between QTL and phenotypes, as well as relationships among phenotypes [15]. Causal edge detection is sensitive to subtle correlation patterns in the data. Inferences have been shown to be subject to a large proportion of false positive edges and can be skewed by environmental and experimental design factors that are not accounted for in the model [16], [17]. Agreement between the graphical model and the true underlying biology is a central goal of systems biology. The topology of networks inferred from xQTL data is often interpreted as a reflection of the underlying biological process - which may be metabolic or regulatory in nature, nonlinear, and involve the dynamic interaction of molecules within cells and tissues. However, the extent to which graphical models derived from static data capture these processes is not well understood, which makes the interpretation of edges challenging.

Deterministic models of cellular metabolism can be defined by ordinary differential equations (ODEs) derived from simple laws of mass-balance [18]–[21]. The reaction rates are modeled as non-linear processes, e.g. Michaelis-Menten kinetics and Hill functions, which depend on kinetic rate parameters [22]. Models of this type are powerful because of their ability to make *in silico* predictions of the response of a system to perturbations. We present a simulation study in which we generate synthetic mQTL data from dynamical models of pathway motifs with two sources of perturbation. We vary the rate parameters in a manner that mimics a genetic cross and we drive the simulations models with an input function that includes stochastic noise.

**Figure 1:**
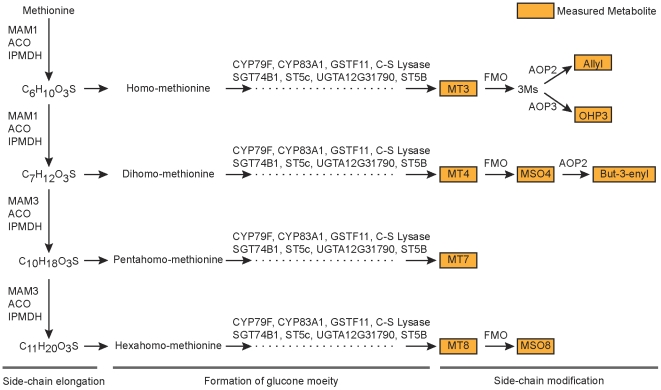
Biosynthesis of aliphatic glucosinolates. The aliphatic glucosinolate biosynthetic pathway occurs in three stages: (1) side chain elongation, (2) formation of glucone moeity and (3) side-chain modification. The metabolites that are measured in the Bay
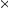
Sha RIL population are indicated together with the facilitating enzymes.

Glucosinolates are secondary metabolites that influence the interaction of plant and pest and have a wide range of important functions in human health [23]–[25]. The economic importance of glucosinolates has led to significant progress in understanding the biochemical pathways and genetics [26], [27]. Glucosinolate biosynthesis occurs in three well understood stages in which amino acids undergo (Figure 1): (1) chain-elongation, (2) formation of glucone moeity, and (3) side-chain modification. In this work, we examine mQTL data from a class of aliphatic glucosinolates in a highly replicated *Arabidopsis* Bay
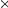
Sha recombinant inbred population [28]. The metabolites under investigation participate in side-chain reactions. Genetic analysis reveals shared QTL and wide-spread epistasis in the pathway [29].

In order to address these questions, we have inferred causal networks from mQTL data using simulated metabolic models of common *pathway motifs* and real data from a well characterized metabolic network. We demonstrate that correlation structure can be shaped by a variety of factors, including, genetic variation, pathway architecture, position in the pathway and feedback. Our results highlight the necessity of biological variation outside of the variation contributed by genetic factors for reliable network inference. Substrate-product relationships are not always reflected in the correlation structure of the system and recovery of the biochemical ordering of species should not be expected. Substrate inhibition, which is pervasive in metabolic pathways, can diminish or mask these relationships and lead to missing edges in network inference. An accurate genetic model is also critical to the inference process, especially when epistasis is involved. Our findings should temper expectations and provide new insights into the interpretation of causal genotype-phenotype networks.

## Results

**Figure 2:**
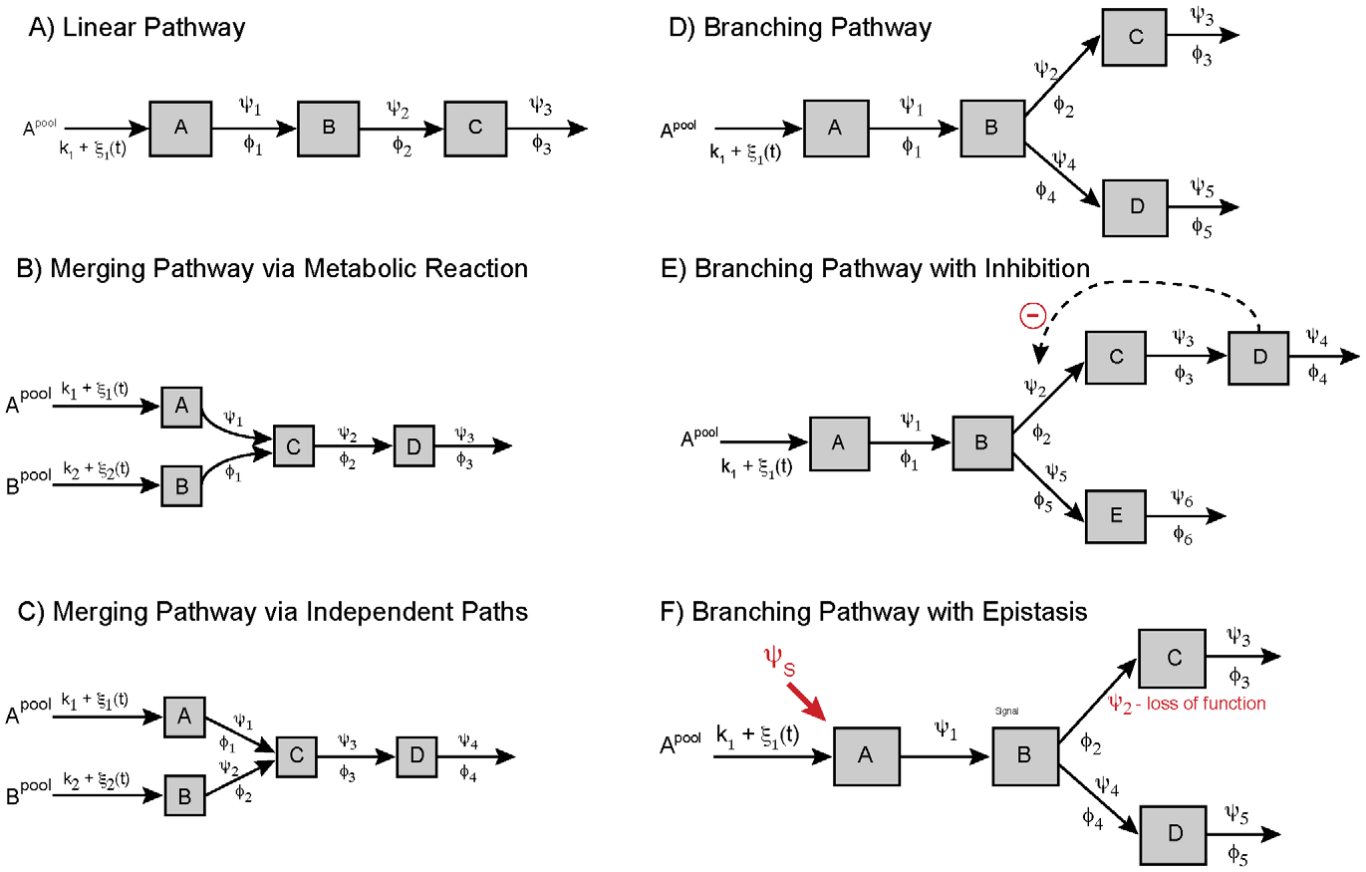
Simulated pathway motifs. (A) Linear, (B) merging pathway via metabolic reaction, (C) merging pathway via independent paths, (D) branching pathway, (E) branching pathway with inhibition, (F) branching pathway with epistasis. 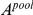 represents a constant pool of metabolite 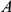 taken up at a constant flux rate 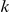 that is subject to a stochastic perturbation 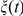, 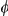 represents the flux rate, 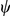 is a genetic perturbation and 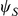 denotes an upstream signal that is affecting the pathway.

Pathway motifs were constructed using ODEs (Figure 2). Flux rates, 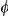, were described with Michaelis-Menton kinetics. Simulations were performed under genetic perturbations, 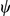, with stochastic input, 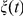 (Figure S1). The aliphatic glucosinolate biosynthetic pathway from an *Arabidopsis* Bay
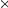
Sha population was also investigated (Figure 1). For each pathway, we carried out a three-step analysis: (1) QTL mapping for the metabolites in the pathway to identify the relevant genetic factors. (2) Metabolite correlations were calculated with and without conditioning on genetic factors. Correlation after conditioning represents the association between metabolites that is driven by sources outside of the genetic factors, e.g., propogation of random input fluctuations through the pathway. Correlation that disappears after conditioning implies an independent relationship between metabolites, e.g., 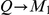 and 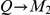. We interpret the presence of correlation after conditioning as being indicative of either causal or reactive relationships, e.g., 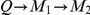 or 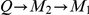. (3) We generated multiple causal networks from their posterior distribution, using a MCMC algorithm previously described [14] and summarized results across the ten top scoring networks.

### Simulated Pathway Motifs

#### QTL detection

**Figure 3:**
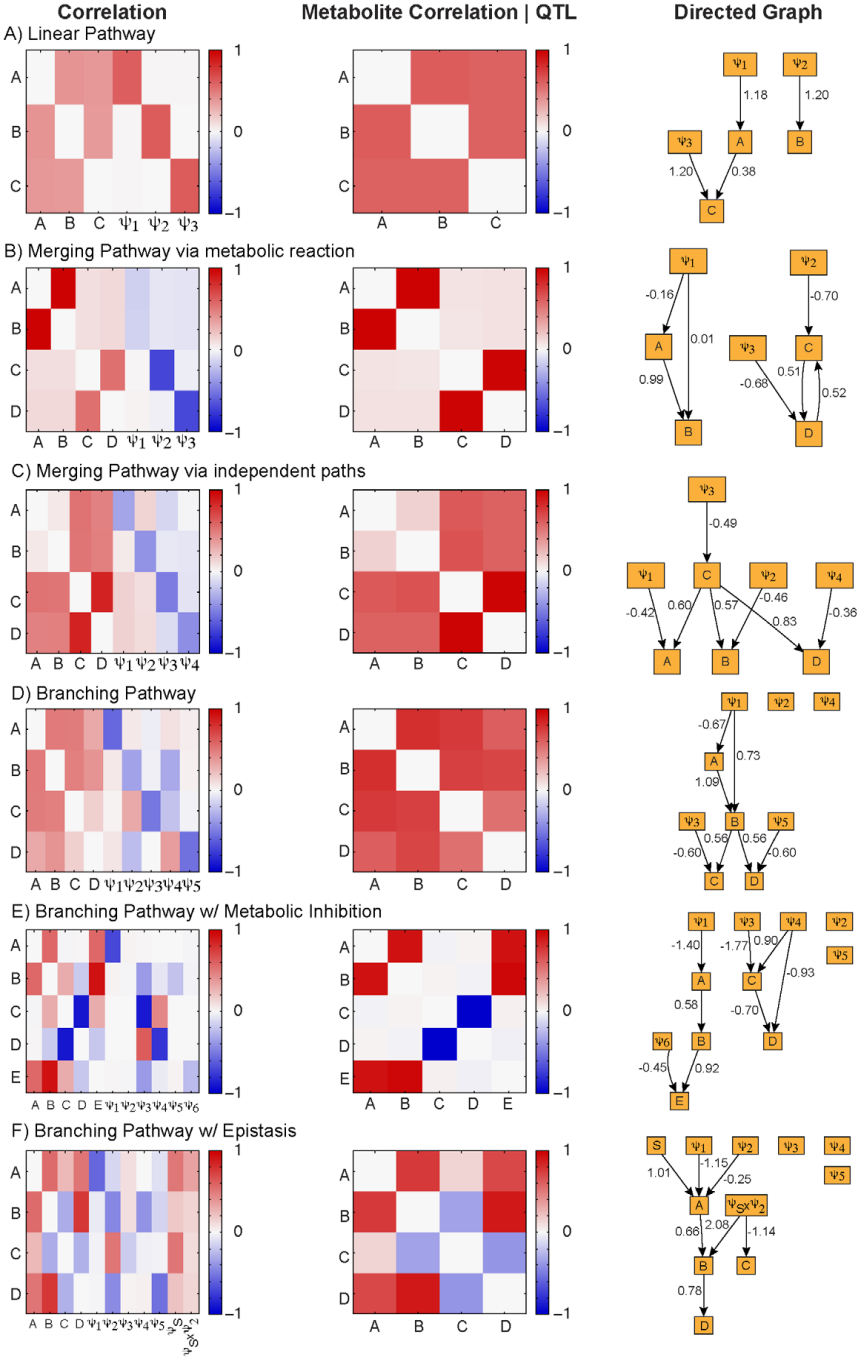
Simulation results. Left: The correlation between metabolites and genetic multipliers, correlation indicates evidence of a QTL, the sign and magnitude indicate direction and size of the effect respectively. Center: metabolite correlation after conditioning on QTL. Right: The inferred causal graphical model estimated from the top ten graphs from MCMC. Edge weights indicate regression coefficients.

Correlation of the genotype variable, 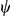, and a metabolite is considered evidence for a QTL with the sign and magnitude indicating the direction of the effect and the effect size (Figure 3). A similar QTL pattern is observed between pathways that contain linear chains of reactions. Specifically, the QTL for a substrate metabolite in a linear chain is the 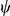 facilitating the downstream flux (e.g., Figure 3A). In the *merging pathway via metabolic reaction*; there are no QTL for the bi-substrate reaction that occurs at the merge point (Figure 3B). However, when the merging pathway is formed through two independent paths QTL mimic the linear pathway pattern (Figure 3C). The QTL effect pattern in the *branching pathway* illustrates the activation of the lower and upper branch (Figure 3C). When the flux through the upper branch is dominant, the production of 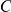 is demanding substrate 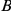, which is then less available for the production of 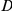. This scenario is reflected in positive correlation between 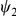 and 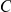, and the negative correlation between 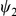 and 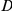 and 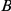. An analogous story plays out for the lower branch and is seen in the 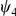 relationships. *Substrate inhibition* in the branching pathway results in the loss of QTL at 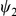 which facilitates the inhibited flux (Figure 3E). In the *branching pathway with epistasis*, 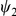 is a QTL for the branch-point metabolite 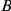, and both 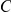 and 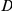 which reside on the branches (Figure 3F). The direction of the effect is a reflection of the metabolite position in the pathway. Epistasis has the strongest effects on 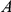 and 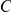 which are immediately downstream of the interacting signal and enzyme respectively.

#### Metabolite correlations

In most cases, the correlation between metabolites after conditioning on genotype variables was enhanced (Figure 3). Substrates in the *linear pathway* are uniformly correlated both before and after conditioning on QTL (Figure 3A). In the *merging pathway via metabolic reaction*, a high correlation between 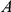 and 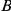 suggests that they must be coordinated to form a product 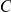 (Figure 3B). In the *merging pathway via independent paths* 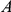 and 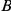 are uncorrelated, 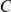 and 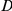 are highly correlated to each other, and to a lesser degree with 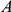 and 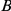 (Figure 3C). In the *branching pathway* 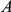 and 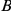 are highly correlated and relationships involving 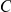 and 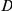 become more pronounced after conditioning (Figure 3D). *Substrate inhibition* is observed in the negative correlation of 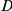 with the other metabolites in the pathway (Figure 3E). The correlation in this pathway was the most sensitive to conditioning on QTL. After conditioning there was almost a total loss of correlation between 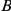 and metabolites on the upper branch, 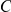 and 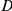 (Figure 3E). In the *branching pathway with epistasis*, 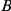 and 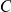 are negatively correlated reflecting the accumulation of 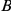 when there is an allelic combination that results in the loss of function of 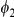 (Figure 3F). The strongest correlation is between 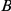 and 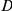.

#### Network reconstructions

The linear and merging pathway reconstructions did not mimic the ordering in the metabolic pathway (Figure 3A–C). A causal edge 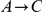 occurred in the *linear pathway* in the ten best scoring models (Figure 3A), but faded when larger subsets of models were considered (Text S1). In the *merging pathway via metabolic reaction* a causal edge 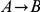 and an undirected edge between 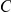 and 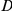 were identified, with no link between the two pathway segments (Figure 3B). When 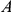 and 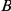 form 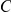 from *merging independent pathways*, 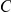 is predicted as a hub metabolite that affects both upstream and downstream neighbors. It is reasonable that 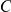, the merging point, controls the influx and efflux of the pathway and dominates the overall correlation structure (Figure 3C). The graphical model for the *branching pathway* captures the biochemistry exactly but does not include the genetic factors (Figure 3D). When *substrate inhibition* occurs in the branching pathway, the graphical model identifies the top and bottom branches, but does not link them together (Figure 3E). In the network reconstruction of the *branching pathway with epistasis*, the lower branch of the pathway is captured exactly and the epistasis term was found to affect 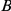 and 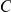 independently (Figure 3F).

### Bay
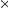
Sha: Aliphatic Glucosinolate Biosynthesis

#### QTL detection

**Figure 4:**
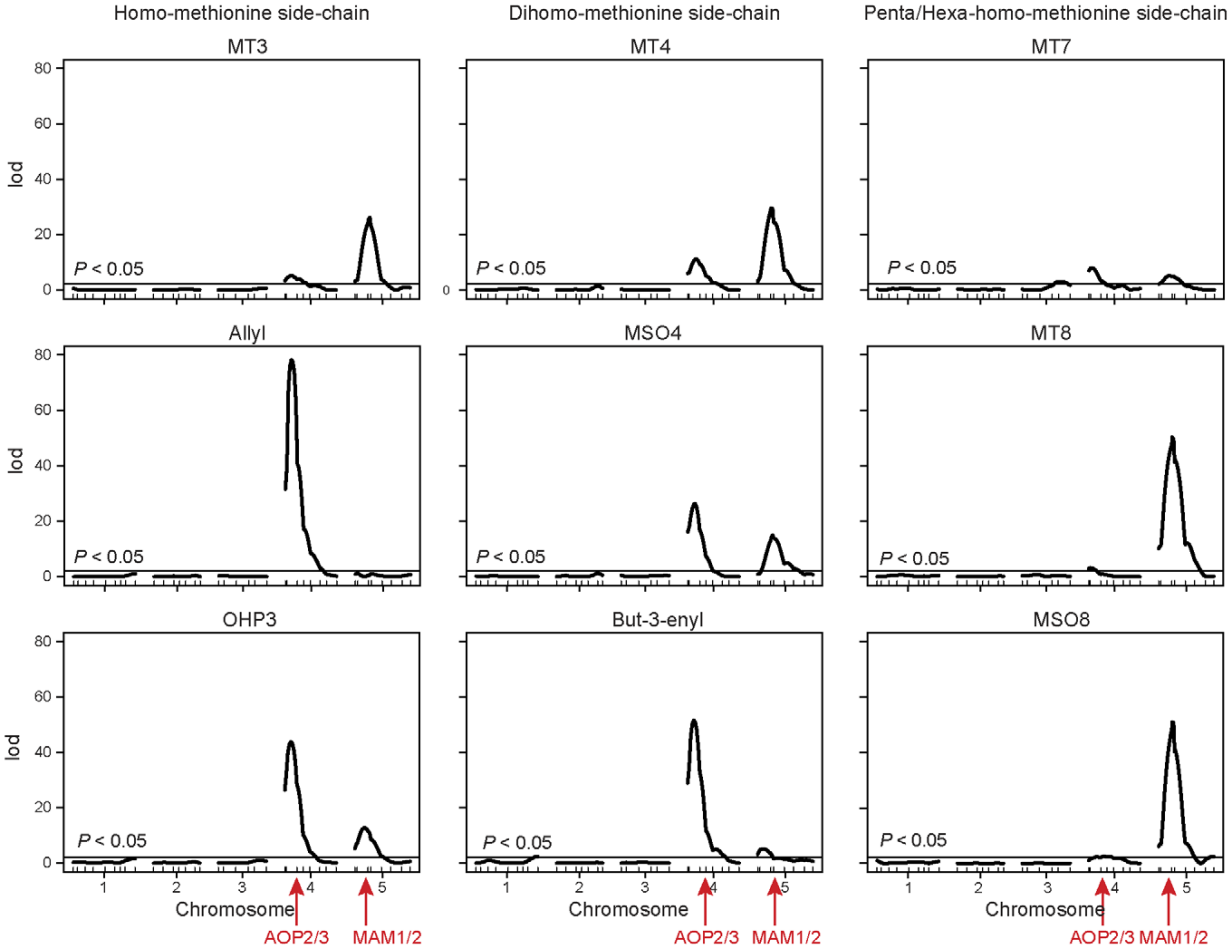
Genome scans for the aliphatic metabolites. QTL mapping was performed for metabolites in the homo-methionine, dihomo-methionine and penta/hexa-methionine side-chains from the Bay
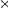
Sha RIL population.

Significant QTL were identified for all of the metabolites in the aliphatic glucosinolate biosynthesis pathway (Figure 4, Tables S1, S2). Common QTL on Chr4 and Chr5 with large effects were detected for most of the metabolites. Two-dimensional genome scans showed a significant epistatic interaction between these two loci, especially in the homo-methionine and dihomo-methionine side chains (Table S3, Figure S2). MT3 showed evidence of two interacting QTL on Chromosome 5. These results are consistent with previous findings [28]. AOP2/3 and MAM1/3 are candidate genes under the QTL peaks on Chr4 and Chr5 respectively [28].

#### Metabolite correlations

**Figure 5:**
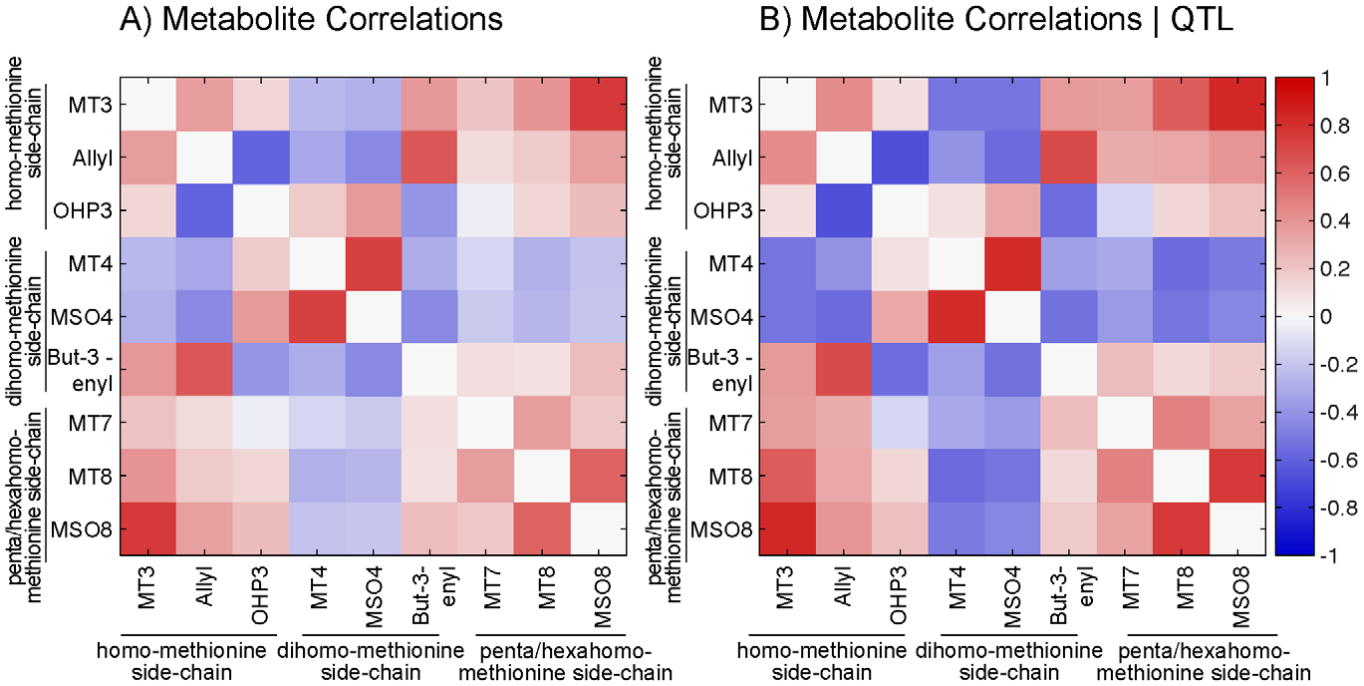
Aliphatic metabolite correlations. Correlation of metabolites in from the Bay
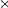
Sha RIL population with (A) no conditioning on QTL and (B) after conditioning on QTL.

Correlation dissipated non-uniformly after conditioning metabolites on QTL (Figure 5). In the homo-methionine pathway, after conditioning, MT3 and Allyl are positively correlated (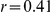), Allyl and OHP3 have a strong negative correlation (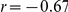), and the correlation between MT3 and Allyl is positive and weaker 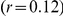. After conditioning in the dihomo-methionine pathway, MT4 and MSO4 are highly correlated (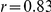), and But-3-enyl is negatively correlated with both Mtb4 and MSO4 (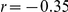 and 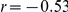 respectively). In the hexahomo-methionine pathway, MT8 and MSO8 are highly correlated (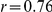) after conditioning. The most profound loss of correlation after conditioning was observed between MT4 and MSO4 and the other metabolites in the pathway with the exception of OHP3. The dramatic reduction indicates that much of the correlation between metabolites is due to shared genetic effects and is not a result of biochemical pathway linkages, consistent with what we know about these pathways.

#### Network reconstructions

**Figure 6:**
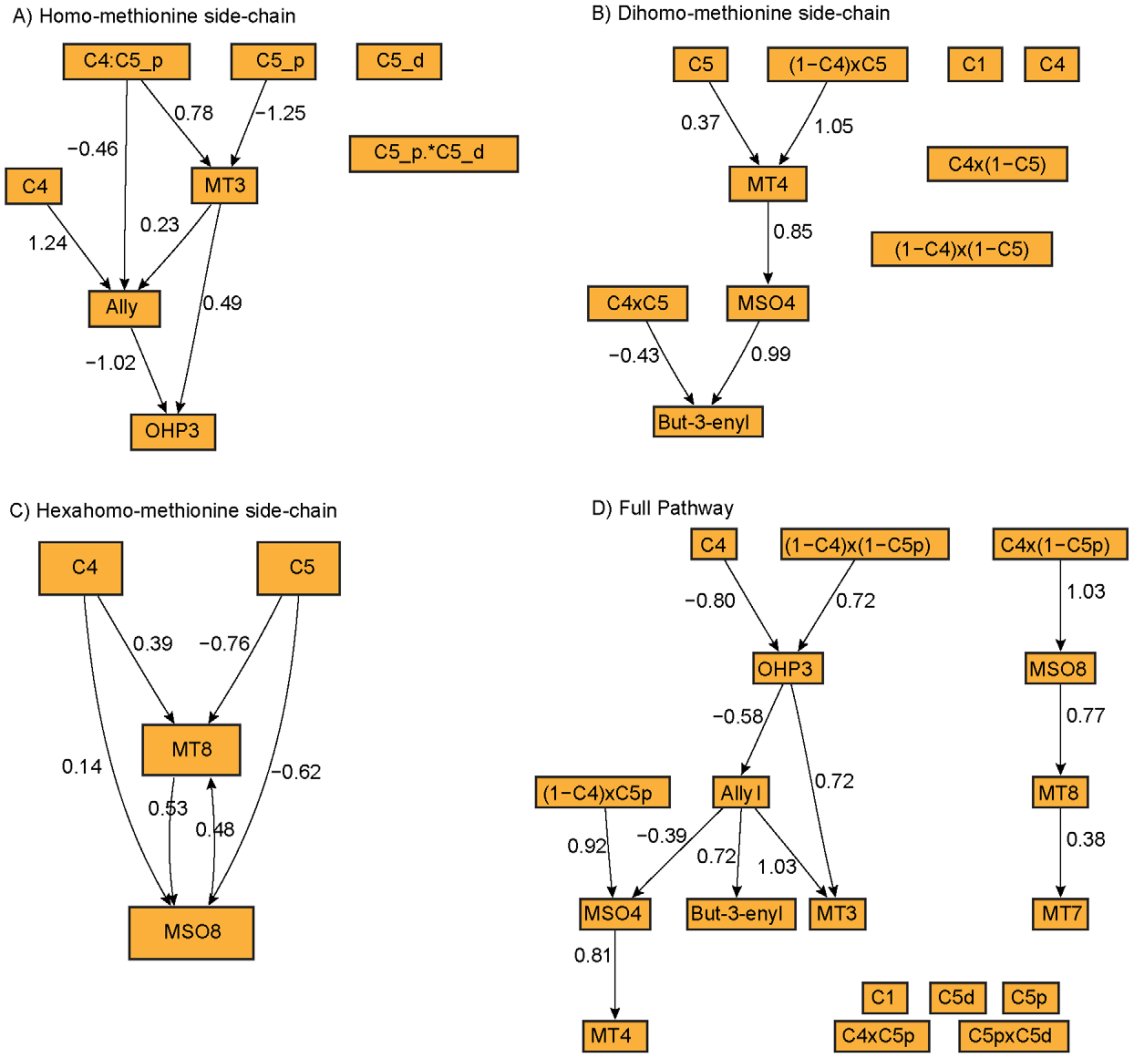
Aliphatic glucosinolate network reconstructions. The (A) homo-methionine, (B) dihomo-methioine and (C) hexahomo-methionine side chains were reconstructed independently. (D) The network was reconstructed from the entire panel of aliphatic metabolites and their QTL. Edge weights indicate regression coefficients.

Side chains: homo-methionine, dihomo-methionine and hexahomo-methionine, were first examined independently (Figure 6A–C). In the homo-methionine reconstruction, the dominant allele at the QTL directly affects Allyl and MT3, and indirectly affects OHP3 through the other metabolites. The order of metabolites in the dihomo-methionine pathway network reconstruction matched the biochemical pathway exactly (Figure 6B). QTL were estimated to directly affect MT4 and But-3-enyl. The hexahomo-methionine chain shows little evidence of epistasis, thus the interaction terms were omitted from the analysis (Figure S2). MT8 and MSO8 were highly correlated, and both have QTL on Chr 4 and 5 with similar effect sizes (Figures 4–5). The graphical model is dense and identifies a connection between MT8 and MSO8, but the direction of causality is not clear (Figure 6C).

The entire panel of QTL and metabolites from the glucosinolate biosynthesis pathway were examined in a single model (Figure 6D). The graphical model groups the top half (homo-methionine and dihomo-methionine side chains) and the lower half (pentahomo-methionine and hexahomo-methionine side-chains). Within these groupings, the side chain members are connected, but the order does not match the biochemical pathway ordering. There is a spurious connection between But-3-enyl and Allyl. Although pathway members grouped together, the direction of causality did not reflect the biological pathway or the ordering inferred for the independent side-chains.

### Propagation of Residual Variance

In order to infer a causal relationship between a substrate 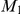 and its product 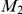, non-genetic variation in substrate concentration has to propagate to the product. This is a necessary, but not sufficient condition for causal inference. To see this, suppose that one metabolite is causal to another, and that variation includes a genetic driver, 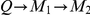. The linear equations for the causal graphical model can be written as:
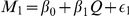


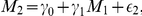
 or equivalently:
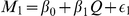


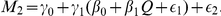



Suppose there is no propagation of the non-genetic variation, 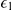, then:
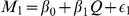


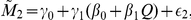
and the traits are conditionally independent given genotype, 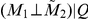. It is clear from the equations that, 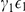 is the term that carries the residual correlation between 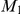 and 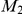. Therefore, variation in metabolites beyond that induced by genotype must be propagated through the biological pathway to create the correlation structure necessary for causal inference.

**Figure 7:**
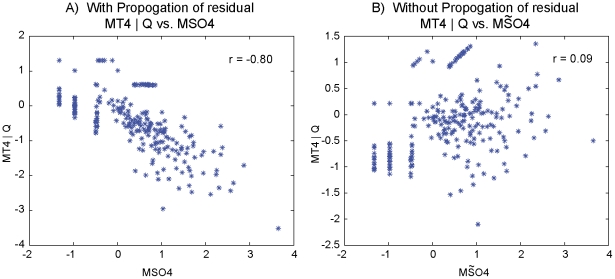
Residual propagation. A real data illustration of the necessity of non-genetic residual propagation for causal inference. Consider the causal model: 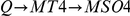, where 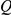 denotes the QTL on Chrs 4, 5 and their interaction. Comparison of 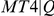 and 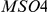 shows correlation suggesting a causal reaction. If the residual variation did not propagate (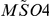) then 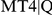 and 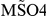 are approximately independent.

Consider the Bay
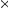
Sha data example: 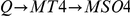, where 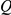 denotes the QTL on Chrs 4, 5 and their interaction. There is a strong correlation between the residuals 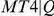 and 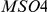 (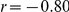) (Figure 7A), which is driven by the propagation of the non-genetic variation, 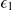. To see this dependency, we imputed data with no propagation of variation:
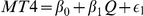


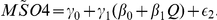


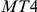
 and 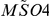 are approximately independent with negligible correlation (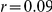). A causal edge between 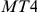 and 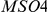 would not be detected with network inference (Figure 7B).

## Discussion

Graphical models provide a framework for estimating causal relationships between genotypes and phenotypes. Models of this type can be used to perform *in silico* experiments that predict responses to genetic and environmental perturbations. Ideally, these models should inform us about of the response to targeted interventions, such as a drug that alters the properties of a metabolic enzyme. There are numerous reasons for caution in such inferences. The inference models are linear, but the true relationships among relevant variables is likely to be driven by a non-linear dynamical process. It is not clear that these relationships should be captured by linear correlation. Correct interpretation is important, particularly if the graphical models are used to guide intervention strategies.

Several algorithms have been proposed for building graphical models in the context of genetic crosses [8]–[14]. These methods all derive models from the correlation and partial correlation structure in the data. We found that the available model building methods produced highly concordant results for models of the size and architectures considered here. Therefore we chose one specific MCMC algorithm to investigate the relationship between an inferred graphical model and the biochemical pathway that gave rise to the data. An advantage of the MCMC algorithm is the ability to sample multiple networks from a posterior distribution. This avoids reliance on a single network, which is problematic when two or more distinct networks can explain the data equally well. Sampling also provides a measure of uncertainty in the inferred network topology. Summarizing an ensemble of networks is challenging. We chose a consensus representation consisting of edges that occur most frequently in the sampled networks. If there is not enough information in the data to reliably establish the existence of an edge, this is reflected in low edge weights of the consensus network. Also, if we observe an edge that is present in most of the sampled networks but with opposing directions in different networks, we can conclude that the edge is present but there is insufficient data to resolve it direction (e.g., Figure 6C).

We analyzed metabolite data and from real and simulated pathways with known network stoichiometry. The Michaelis-Menton kinetics used in our simulated metabolic reactions are special cases of Hill functions and represent a rough approximation to actual enzyme reactions. Similar models have been used to describe gene regulatory networks and other biological phenomena, e.g. [19], [20], [30]. Constraint based modeling provide an alternative approach to delineate metabolic networks from steady-state data [31]. In the steady-state, the system of ODEs reduces to a linear system, but nonlinear relationships may arise between fluxes and pathways [32]. Investigation of the properties of constraint based and other non-correlation based methods for inference in dynamical systems remains an area of active research [33]–[36].

Correlation in metabolite data can be driven by a variety of factors that do not directly relate to the network stoichiometry. In order to capture the biochemical ordering of the pathway, noise has to propagate through the biochemical network. Many biological pathways are buffered by feedback or other stabilizing features that reduce noise propagation and mask the correlations that would imply causal connections. Failure to consistently observe substrate-product correlation may explain some of the differences observed between the plant data and simulations for matching pathway architectures. Our objective is not to confirm that our simulations accurately reflect the plant data or to make generalizations about certain pathway architectures. Rather, we seek to leverage real data from a well-studied biological system and simulated data from pathway motifs to explore a variety of architectures and conditions. A shortcoming of *in silico* models is their inability to fully capture the richly interconnected nature of biological systems. We considered simple motifs in isolation and modeled them with Michaelis-Menton kinetics. Correlation structure depends on the network architecture, the size and nature of the genetic perturbation, stochastic fluctuation, and enzyme kinetics. The advantage of this simulation is that no biological variation arises from factors outside of what is modeled. Whereas, metabolic systems *in vivo* contain mechanisms that make them robust, e.g., buffering, cycling and feedback, but may be impossible to pin-point with real data.

In the plant data, many of the substrate-product relationships remain intact after conditioning on QTL (Figure 5). This suggests that a real metabolic pathway may give rise to meaningful biological correlations that reflect the topology of the pathway despite the non-linear nature of the underlying processes. This is promising from the point of view of network reconstruction, but is not without limitation. The architecture of the homo-methionine side-chain was only partially captured, with an additional edge between Allyl and OHP3 that reflects the shunting of flux through the lower branch of the pathway (Figure 6A). The biochemical ordering of the dihomo-methionine side-chain was captured exactly (Figure 6B). We are only to able to detect an undirected connection between MT8 and MSO8 in the hexahomo-methionine side-chain (Figure 6C). Lack of a private QTL or a gradient in the effect size gives rise to likelihood equivalent models from which the direction of causality could not be distinguished. A similar situation was observed when a global model was estimated from the entire panel of metabolites and QTL (Figure 6D). The shared nature of the QTL hindered network reconstruction of the entire pathway. Most of the side-chain members were linked, but the direction of causality was not consistent with the pathway or with the networks constructed for each of the side-chains independently. Allyl and But-3-enyl are unlinked in the metabolic pathway, but are both products in reactions facilitated by AOP2. The causal link between them is likely driven by this co-regulation.

Conditioning on QTL genotypes strengthens the correlation among metabolites in most of the simulated pathway motifs (Figure 3). An exception occurs in the branching pathway with substrate inhibition which shows an almost complete loss of correlation between the branchpoint 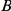 and upper branch metabolites 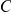 and 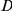 after conditioning (Figure 3F). In the linear pathway, when reaction rates are not operating at saturation and there are no branches to redirect the flux, any variation in the flux must propagate through each of the metabolites [37]. This results in a uniform correlation structure among the metabolites, which in turn yields weak causal linkages and order ambiguity among metabolite nodes in the graphical model. However, graphical models strongly and consistently associate metabolites to the QTL node controlling their downstream flux in linear pathways (Figure 3A, Text S1). The branching pathway is a linear pathway with a sink that represents demand on a metabolite from another reaction or pathway (Figure 2D). The stoichiometry of the branching pathway was captured exactly with the graphical model (Figure 3D). This suggests that the diversion of flux through side reactions is helpful in defining pathway order. For merging pathways, the correlation structure is dependent on the nature of the reaction at the merge point. When two pathways merge through a bi-substrate reaction (Figure 2B) there is strong association between the substrates that combine, but these are only weakly coupled to the downstream component of the pathway. On the other hand, when two pathways merge through independent reactions, the upstream metabolites 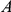 and 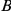 are only weakly correlated with each other, but the there is strong uniform correlation across the two linear components of the pathway (Figure 3C). Ordering metabolites in the independent merging pathway suffers from the same weaknesses as in the linear pathway. These results emphasize the influence of network stoichiometry on the correlation structure of the pathway.

Biosynthetic pathways, which often branch to produce two or more end products, are especially prone to inhibition [38]. We examined biosynthetic pathways that were inhibited in two ways: (1) loss of function in one pathway branch and (2) substrate inhibition. In the plant data, loss of function in AOP2 gave rise to an epistatic interaction between loci on Chr 4 and Chr 5 [28], [29]. Ignoring epistatic interactions and model fitting with only main-effect terms led to dense graphs that were difficult to interpret (data not shown). Substrate inhibition is estimated to occur in approximately 20% of enzymes [39]. This process can be viewed as a regulatory mechanism in which accumulation of a substrate represses the reaction velocity. In our simulation, the accumulation of metabolite 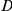 inhibits the flux through a branched pathway (Figure 2E). The inhibition is reflected in the correlation structure, 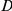 is negatively correlated with the other metabolites (Figure 3E). QTL 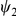 disappears, suggesting that substrate inhibition can dominate the effects of genetic perturbations (Figure 3D–E). The correlation structure of this pathway was most sensitive to conditioning on QTL. When substrate inhibition is present, a loss of correlation and genetic control can occur, which makes two connected pathways look independent. These results highlight the importance of an accurate genetic model for network inference, especially in the presence of inhibition and epistasis.

Estimation of kinetic parameters in dynamic models requires time course data, which is often sparse, and the computations involved can be challenging [40]. The choice of experimental perturbations and design have been shown to have major influence on parameter estimation, and subsequently the accuracy of the computational model [41]. Complex models of biological systems exhibit parameter sensitivities that span several orders of magnitude [42]. Concentration profiles and model outputs are sensitive to small changes in kinetic rate parameters [43], [44]. The impact of parameter values on concentrations carries over into the correlation structure, and consequently, the downstream network inference. In our simulations, the perturbation is analogous to genetically determined non-competitive inhibition, where 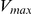 is genetically perturbed to be either *high* or *low*, thereby changing the flux capacity [45]. This strategy ensures that there is a significant difference between genotype groups and enables us to identify QTL. Random stochastic fluctuations were used as input and propagated through the pathway. Stochastic inputs allow us to examine the out of equilibrium dynamics of the system. The fluctuations themselves represent some of the randomness the pathway encounters from being part of a cellular system that is continuously changing [46], [47]. The models represent continuous excitation of the cell with the assumption that the intra-cellular dynamics can be faithfully modeled with ODEs. Examining system behavior over a spectrum of parameter values and stochastic inputs would offer additional insight into the sensitivity of the correlation structure.

Using both real data and simulated data, we tested the ability of graphical models to capture causal relationships between variables from from a variety of metabolic pathway topologies and conditions. We found that the use of linear statistical models to approximate relationships in dynamic non-linear systems from static data has some merit, but the results should be interpreted carefully. It is not realistic to expect to fully recover ordered pathway relationships with causal inference methods. Our results emphasize the necessity of biological variation beyond the genetic factors in the model for reliable network inference. We demonstrated that residual correlation induced between substrate and product in a metabolic reaction can be dominated by variety of factors, including, flux shunting, co-regulation, position in the pathway, genetic factors and inhibition. We found that inhibition can lead to missing edges in graphical models, washing out the genetic signal and making connected pathways look independent. An accurate genetic model is important, especially when epistasis is present. Taken together, these results temper our expectations and explain some of the success and failures of causal graphical models for genotype-phenotype inference.

## Materials and Methods

### Arabidopsis Bay
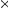
Sha RIL

Metabolic QTL data from a population of 403 *Arabidopsis* Bay
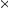
Sha recombinant inbred lines (RIL) were examined in this study [28]. The data include measurements of 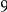 aliphatic metabolites and genotypes from 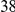 markers across the genome. A substantial number of samples have metabolite levels that are below the level of detection (Table S1). We applied a transformation to the scale 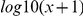. QTL mapping was performed for each metabolite with R/qtl [48]. Genome scans for single-QTL and two-QTL models were performed with Haley-Knot regression. The logarithm of odds (LOD) threshold for significance (*P* 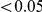) was calculated from 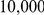 permutations [49].

### Simulating Deterministic Pathway Models

Pathway motifs were used to define systems of ODEs that depend on flux rates, 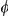, modeled with Michaelis-Menten kinetics (Figure 2) [22]. If a substrate 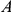 produces 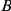, then the rate of reaction 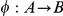 is described by:
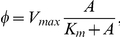
where 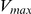 is the maximum rate of velocity and 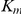 is substrate concentration at which half of 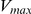 is attained. When two substrates 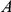 and 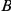 combine to produce 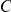, 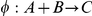, we write:
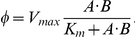
When the accumulation of a metabolite feeds-back to inhibit a flux:
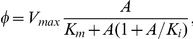
where 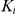 is an affinity constant. This flux form represents *substrate inhibition* which occurs at high substrate concentrations. As 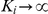, the reaction flux is uninhibited and approaches standard Michalis-Menton form.

The dynamics of a substrate 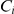 is described with the mass balance equations:
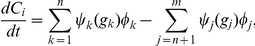
where 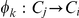 and 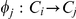 denote the production and utilization of 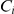 respectively, the stoichiometric coefficients are given as 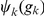 and 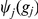 and 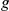 is the genotype. Genetic perturbations are made through the 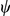 coefficients as either *high* or *low*, depending on the genotype 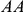 or 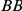. For simplicity, we assume that each 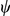 participates in a single reaction and that they are unlinked. In our simulations, we set 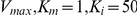, 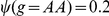 and 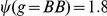. We also modeled a loss of function mutation by setting 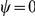 for certain genotypes (Figure S3) [50].

There are 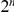 genotype combinations for each pathway of 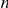 reactions. Each combination can be viewed as a sample from a randomized genetic population. For every unique genotype combination, we use an input flux that is perturbed by a random process, 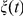, modeled as a Brownian path over the interval 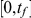 [51]. The system is propagated, 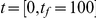. The perturbations, 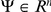, and the concentration levels at the end of the simulation 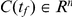 are collected as data for correlation analysis and graphical model fitting. The output of each simulation can be viewed a sample in mQTL data. A schematic depicting the entire simulation process is shown in Figure S1.

### Correlation Analysis and Causal Network Inference

The Pearson correlation is calculated for the variables in each pathway architecture. Residuals are estimated after each metabolite is conditioned on the QTL in the model. The residuals are used to calculate the conditional correlation of the metabolites given the genetic factors in the model. Directed graphical models are estimated using Bayesian Networks with a MCMC algorithm [14]. In pathways with epistasis, we include single degree of freedom variables that represent a composite genotype as variables for inference [52]. The sparsity parameter 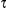 was set in the range 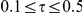. Each chain was run from two starting points, convergence was verified using correlation of edge weights (posterior probabilities) and the acceptance rate of each chain was in the range of 23%–45%. The results are based on the marginal summary over the ten graphs with the highest posterior probability. Alternative representations over the top 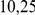 and 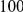 graphs and the four most probable graphs for each pathway are presented in Text S1.

## Supporting Information

Figure S1 **A schematic of the simulation process.** (1) A mathematical model is constructed and described by ODEs, (2) The system is genetically perturbed and propogated. The output of the simulation serves as data for graphical model construction. (3) The correlation structure is observed and graphical models are constructed. The resulting correlation and inferred network is compared to the metabolic pathway.
(TIF)

Figure S2
**Simulated branching pathway with epistasis.** The signal 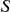 interacts with an enzyme 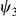 which causes a loss of function for certain genotype combinations.(TIFF)

Figure S3 **Chr4:Chr5 interaction plots.** Interaction plots are shown for each phenotype in the aliphatic glucosinolate pathway.
(TIFF)

Table S1 **Aliphatic metabolites.** Abbreviations and the number of lines that had measurements below detection level are indicated. Non-detection may be due to biological or technical reasons.
(PDF)

Table S2
**Summary of single-locus genome scans for aliphatic glucosinolates.** The chromosome, position, locus, LOD score and peak marker are indicated for each QTL. A significance level of LOD = 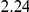 (*P* 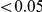) was calculated from 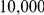 permutations.(PDF)

Table S3 **Summary of two-locus genome scans for aliphatic glucosinolates.** Summary of two-locus genome scans for the metabolites measured in the Bay×Sha RIL panel. Two dimensional genome scans were performed to identify significant interactions. For each pair of chromosomes, the following LOD scores are calculated. **lod.full**: The difference in the maximum LOD score for the full model (two main effect terms and interaction) and the maximum LOD score for the additive model (main effect terms only). **lod.fv1**: The difference in the maximum LOD score for the full model and the maximum LOD score for the LOD score from a single-QTL mapping of the two chromosomes. **lod.add**: The maximum additive LOD score. **lod.av1**: The difference between the maximum additive LOD score and the maximum LOD score from a single-QTL mapping of the two chromosomes. The positions for the full and additive models (pos.f and pos.a respectively) are indicated. Significance thresholds were set at the R/qtl suggested values for a backcross.
(PDF)

Text S1 **Graphical models were reconstructed using a MCMC algorithm.** The result is an ensemble of graphs, each with a posterior probability. Here we present different summarizations of the Bay×Sha reconstructed networks based on model selection and marginal summaries over the most probable graphs.
(PDF)
