# Supplementary figures and images for: What Can Causal Networks Tell Us about Metabolic Pathways?
Source: PLoS Comput Biol. 2012 Apr 5;8(4):e1002458. doi: 10.1371/journal.pcbi.1002458 (PMC3320578; doi:10.1371/journal.pcbi.1002458)

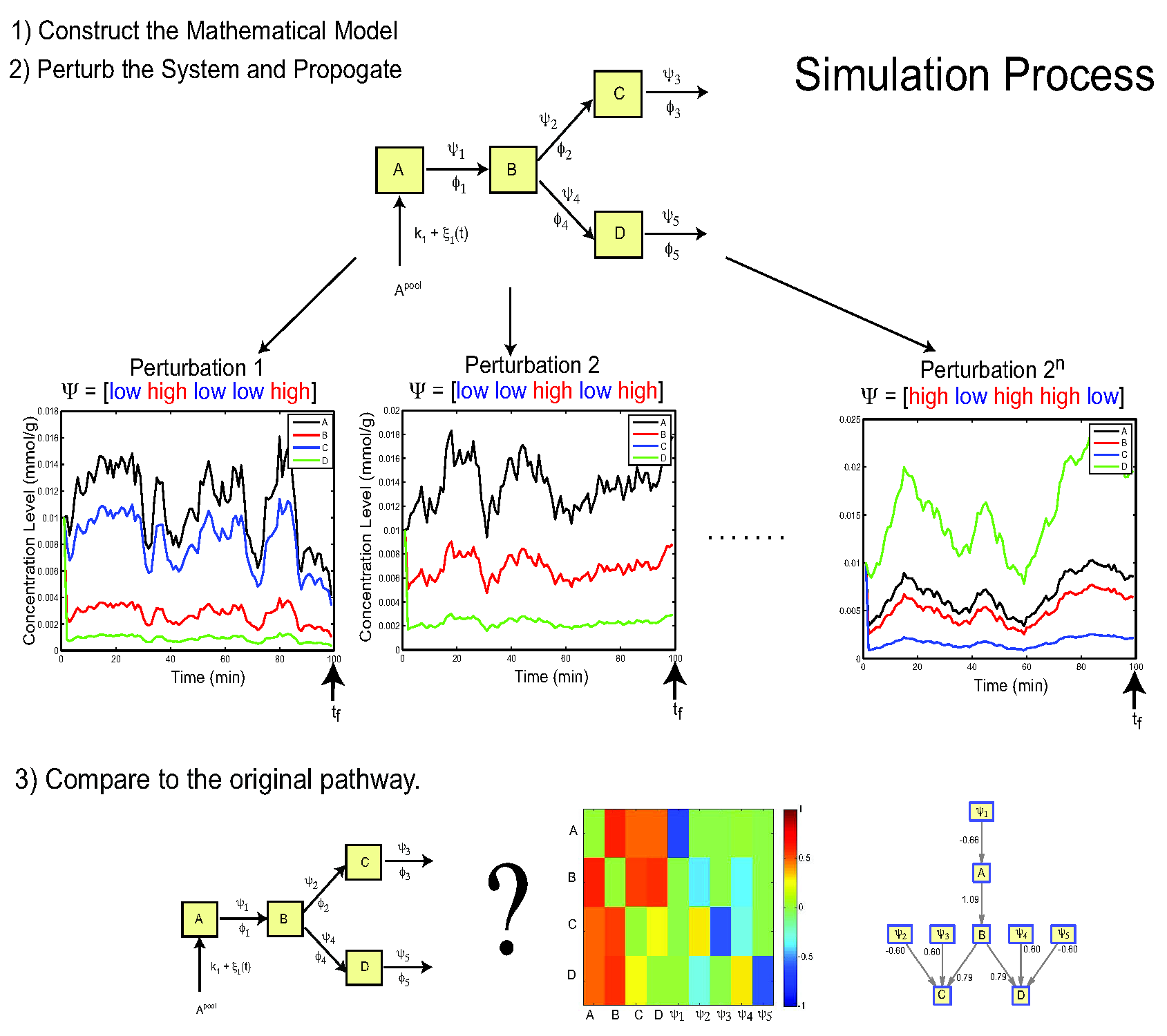

Supplement: Figure S1 — A schematic of the simulation process. (1) A mathematical model is constructed and described by ODEs, (2) The system is genetically perturbed and propogated. The output of the simulation serves as data for graphical model construction. (3) The correlation structure is observed and graphical models are constructed. The resulting correlation and inferred network is compared to the metabolic pathway. (TIF) [file pcbi.1002458.s001.tif]

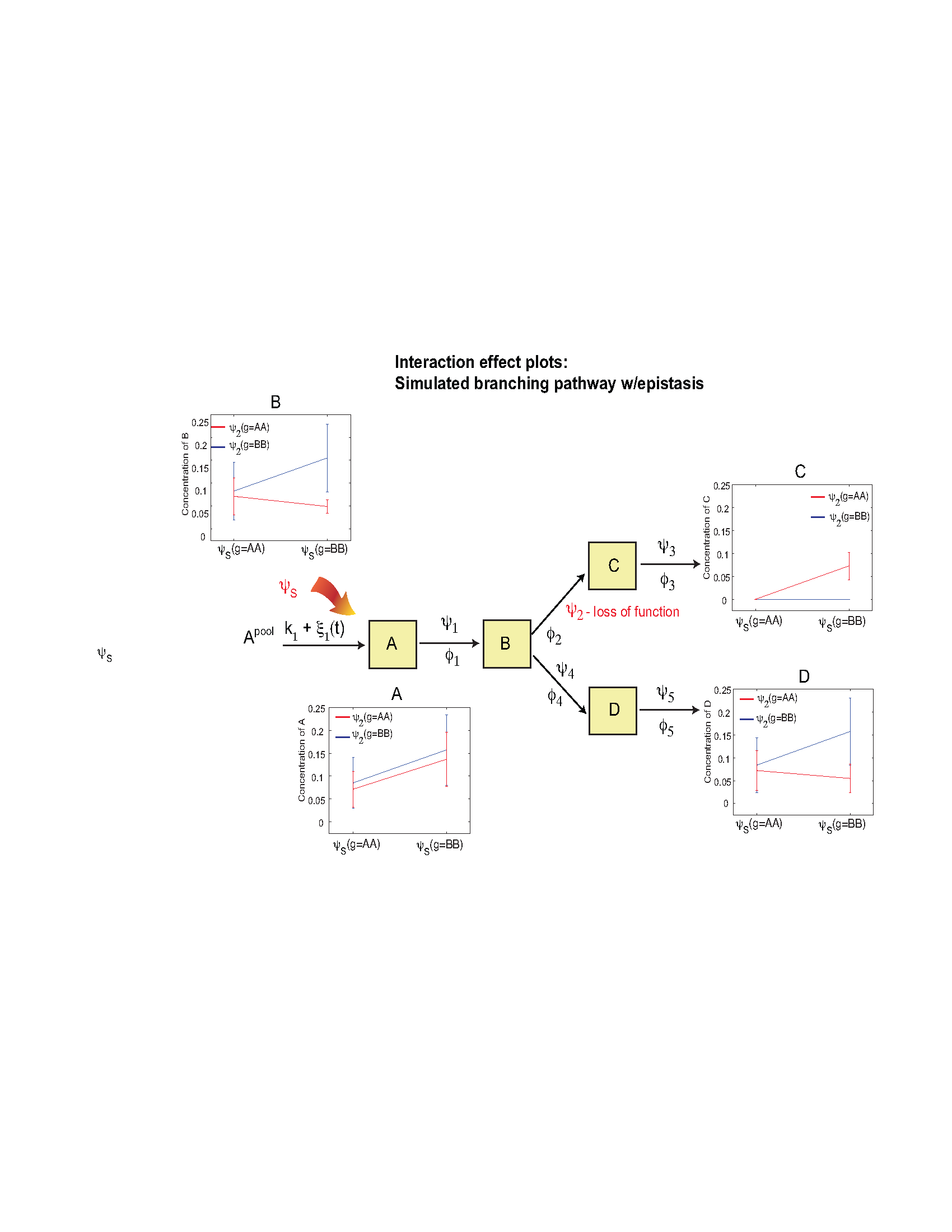

Supplement: Figure S2 — Simulated branching pathway with epistasis. The signal interacts with an enzyme which causes a loss of function for certain genotype combinations. (TIFF) [file pcbi.1002458.s002.tif]

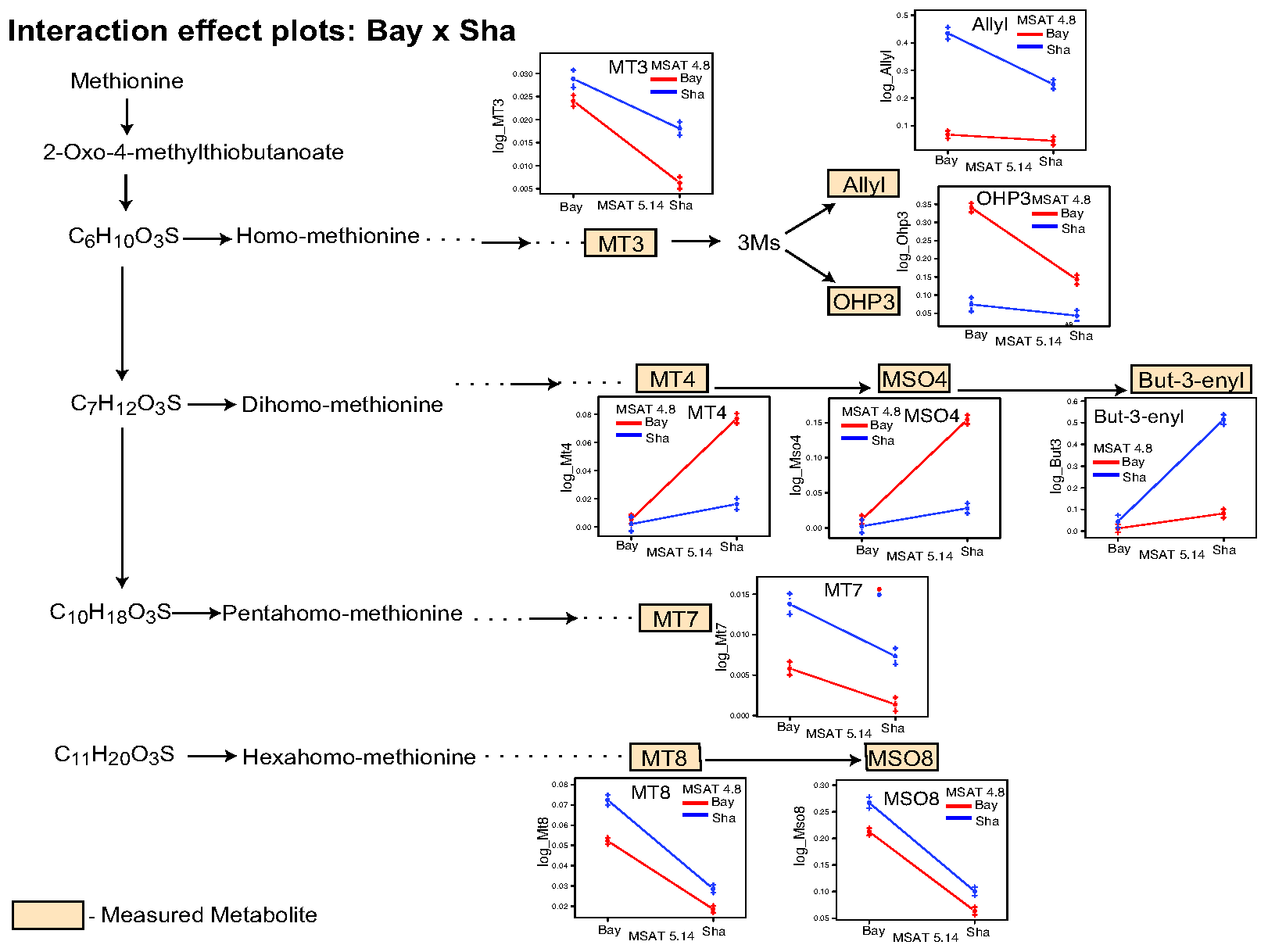

Supplement: Figure S3 — Chr4:Chr5 interaction plots. Interaction plots are shown for each phenotype in the aliphatic glucosinolate pathway. (TIFF) [file pcbi.1002458.s003.tif]
