# Supplementary material for: What Can Causal Networks Tell Us about Metabolic Pathways?
Source: PLoS Comput Biol. 2012 Apr 5;8(4):e1002458. doi: 10.1371/journal.pcbi.1002458 (PMC3320578; doi:10.1371/journal.pcbi.1002458)
Supplement: Table S1 — Aliphatic metabolites. Abbreviations and the number of lines that had measurements below detection level are indicated. Non-detection may be due to biological or technical reasons. (PDF) [file pcbi.1002458.s004.pdf]

Table S1: Aliphatic metabolites in the Bay ×Sha population

Phenotype	Abbreviation	# of lines below detection level
3-Methylthiopropyl-glucosinolate	MT3	45
Allyl-glucosinolate	Allyl	201
3-Hydroxypropyl-glucosinolate	OHP3	7
4-Methylthiobutyl-glucosinolate	MT4	203
4-Methylsulphinybutyl-glucosinolate	MSO4	183
3-Butenyl-glucosinolate	But-3-enyl	158
7-Methylthio-heptyl-glucosinolate	MT7	230
8-Methylthio-octyl-glucosinolate	MT8	1
8-Methysulphinyl-octyl-glucosinolate	MSO8	0
