# Supplementary material for: What Can Causal Networks Tell Us about Metabolic Pathways?
Source: PLoS Comput Biol. 2012 Apr 5;8(4):e1002458. doi: 10.1371/journal.pcbi.1002458 (PMC3320578; doi:10.1371/journal.pcbi.1002458)
Supplement: Table S2 — Summary of single-locus genome scans for aliphatic glucosinolates. The chromosome, position, locus, LOD score and peak marker are indicated for each QTL. A significance level of LOD = (P ) was calculated from permutations. (PDF) [file pcbi.1002458.s005.pdf]

Table S2: Significant QTL: Bay ×Sha metabolites

Pheno	Chr	Pos	LOD	locus	PeakMarker
MT3	4	10	5.497	c4.loc10	NGA8
MT3	5	20	26.972	c5.loc20	NGA139
MT3	5	18	30.943	c5.loc18	MSAT5.14
Allyl	4	8	80.638	c4.loc8	MSAT4.8
OHP3	4	8	45.436	c4.loc8	MSAT4.8
OHP3	5	14	13.164	c5.loc14	MSAT5.14
MT4	4	12	11.865	c4.loc12	NGA8
MSO4	4	10	27.995	c4.loc10	NGA8
MSO4	5	20	14.999	c5.loc20	NGA139
But-3-enyl	1	82	2.317	c1.loc82	MSAT1.5
But-3-enyl	4	8	54.655	c4.loc8	MSAT4.8
But-3-enyl	5	8	5.02	c5.loc8	NGA249
MT7	3	52	3.083	c3.loc52	MSAT3.21
MT7	4	4	7.759	c4.loc4	MSAT4.8
MT7	5	14	5.376	c5.loc14	MSAT5.14
MT8	4	2	3.381	c4.loc2	MSAT4.8
MT8	5	18	55.064	c5.loc18	MSAT5.14
MSO8	4	20	2.681	c4.loc20	NGA8
MSO8	5	18	54.978	c5.loc18	MSAT5.14
