# Supplementary material for: What Can Causal Networks Tell Us about Metabolic Pathways?
Source: PLoS Comput Biol. 2012 Apr 5;8(4):e1002458. doi: 10.1371/journal.pcbi.1002458 (PMC3320578; doi:10.1371/journal.pcbi.1002458)
Supplement: Table S3 — Summary of two-locus genome scans for aliphatic glucosinolates. Summary of two-locus genome scans for the metabolites measured in the Bay×Sha RIL panel. Two dimensional genome scans were performed to identify significant interactions. For each pair of chromosomes, the following LOD scores are calculated. lod.full: The difference in the maximum LOD score for the full model (two main effect terms and interaction) and the maximum LOD score for the additive model (main effect terms only). lod.fv1: The difference in the maximum LOD score for the full model and the maximum LOD score for the LOD score from a single-QTL mapping of the two chromosomes. lod.add: The maximum additive LOD score. lod.av1: The difference between the maximum additive LOD score and the maximum LOD score from a single-QTL mapping of the two chromosomes. The positions for the full and additive models (pos.f and pos.a respectively) are indicated. Significance thresholds were set at the R/qtl suggested values for a backcross. (PDF) [file pcbi.1002458.s006.pdf]

Table S3: Summary of two-locus genome scans: Bay × Sha metabolites

Metabolite	chr1	chr2	pos1f	pos2f	lod.full	lod.fv1	lod.int	pos1a	pos2a	lod.add	lod.av1
MT3	4	5	6.00	20.00	28.71	9.40	1.94	6.00	18.00	26.77	7.46
MT3	5	5	20.00	62.00	23.39	4.08	0.26	20.00	60.00	23.12	3.82
Allyl	4	5	8.00	18.00	73.93	13.89	7.11	10.00	18.00	66.82	6.78
OHP3	4	5	8.00	20.00	66.95	31.71	10.16	10.00	20.00	56.79	21.55
MT4	4	5	10.00	18.00	66.56	42.76	20.13	10.00	18.00	46.43	22.63
MSO4	4	5	8.00	18.00	72.96	47.38	21.74	8.00	18.00	51.22	25.64
But-3-enyl	4	5	10.00	18.00	85.49	51.40	31.37	8.00	18.00	54.12	20.03
MT7	4	5	4.00	12.00	14.53	7.08	0.15	4.00	12.00	14.38	6.92
MT8	4	5	2.00	18.00	63.50	12.38	1.38	2.00	18.00	62.12	11.01
