# Supplementary material for: What Can Causal Networks Tell Us about Metabolic Pathways?
Source: PLoS Comput Biol. 2012 Apr 5;8(4):e1002458. doi: 10.1371/journal.pcbi.1002458 (PMC3320578; doi:10.1371/journal.pcbi.1002458)
Supplement: Text S1 — Graphical models were reconstructed using a MCMC algorithm. The result is an ensemble of graphs, each with a posterior probability. Here we present different summarizations of the Bay×Sha reconstructed networks based on model selection and marginal summaries over the most probable graphs. (PDF) [file pcbi.1002458.s007.pdf]

Text S1: Graphical models were reconstructed using a MCMC algorithm. The result is an ensemble of graphs, each with a posterior probability. Here we present different summarizations of the Bay x Sha reconstructed networks based on model selection and marginal summaries over the most probable graphs.

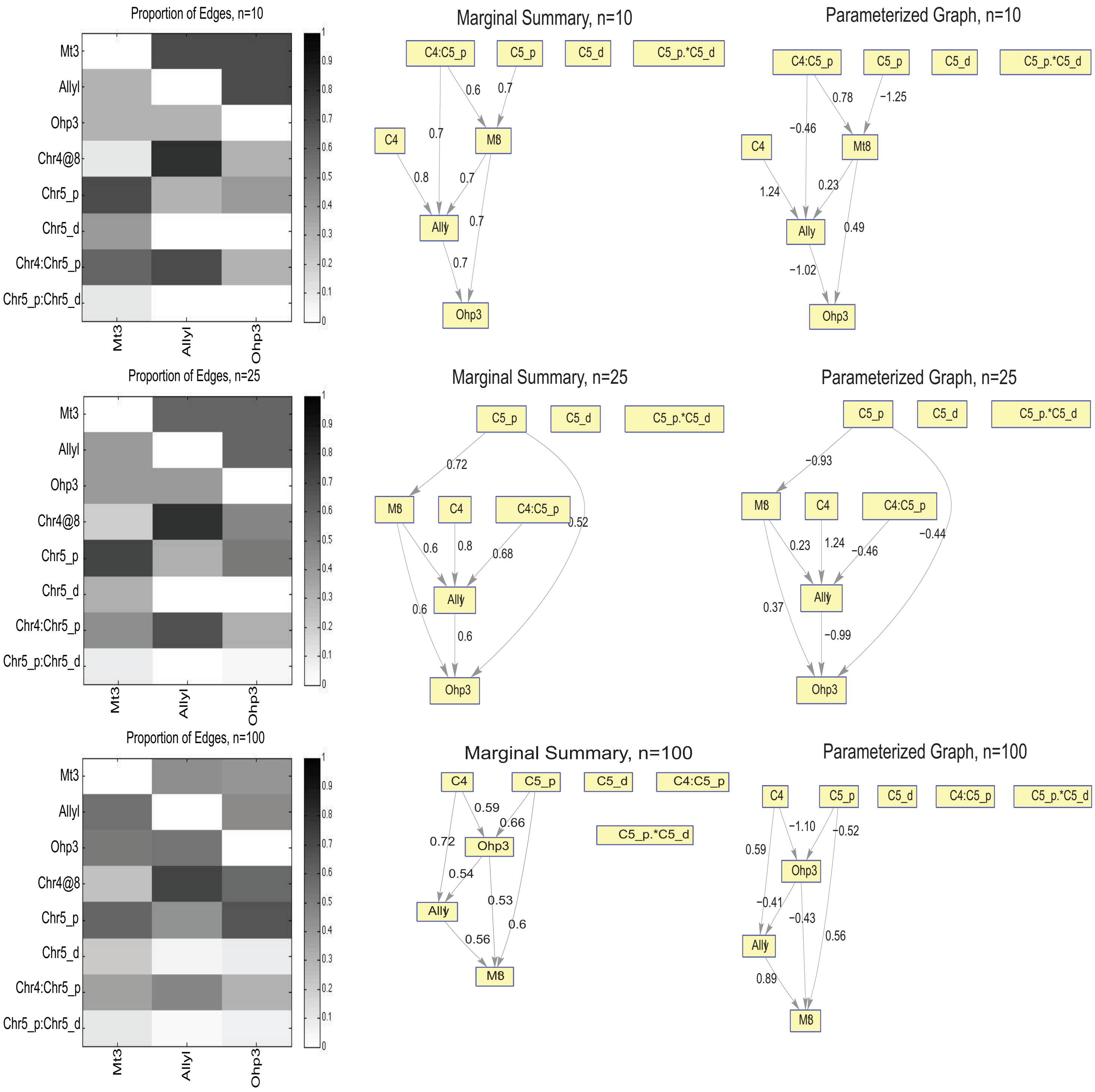

Figure 1. Homo-methionine sidechain network reconstructions. The results are shown for the top scoring $n = 10$, 25 and 100 graphs (top - bottom) identified through MCMC sampling. For each n the proportion of edges present in the summary, marginal summary based on edge proportion of at least 0.5 and the parameterized model is shown (left - right).

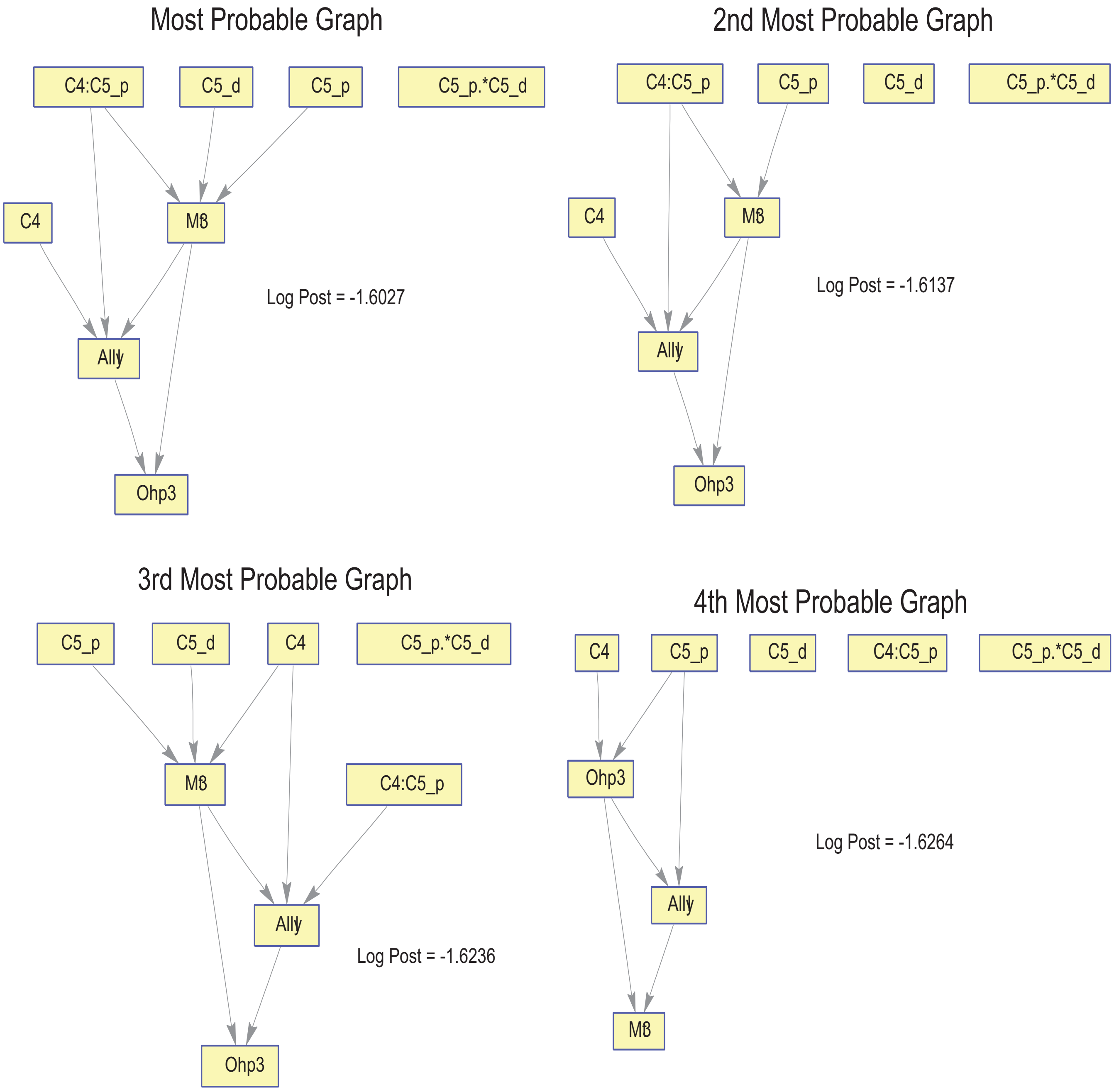

Figure 2. Homo-methionine sidechain top four parameterized network reconstructions and their posterior probabilities.

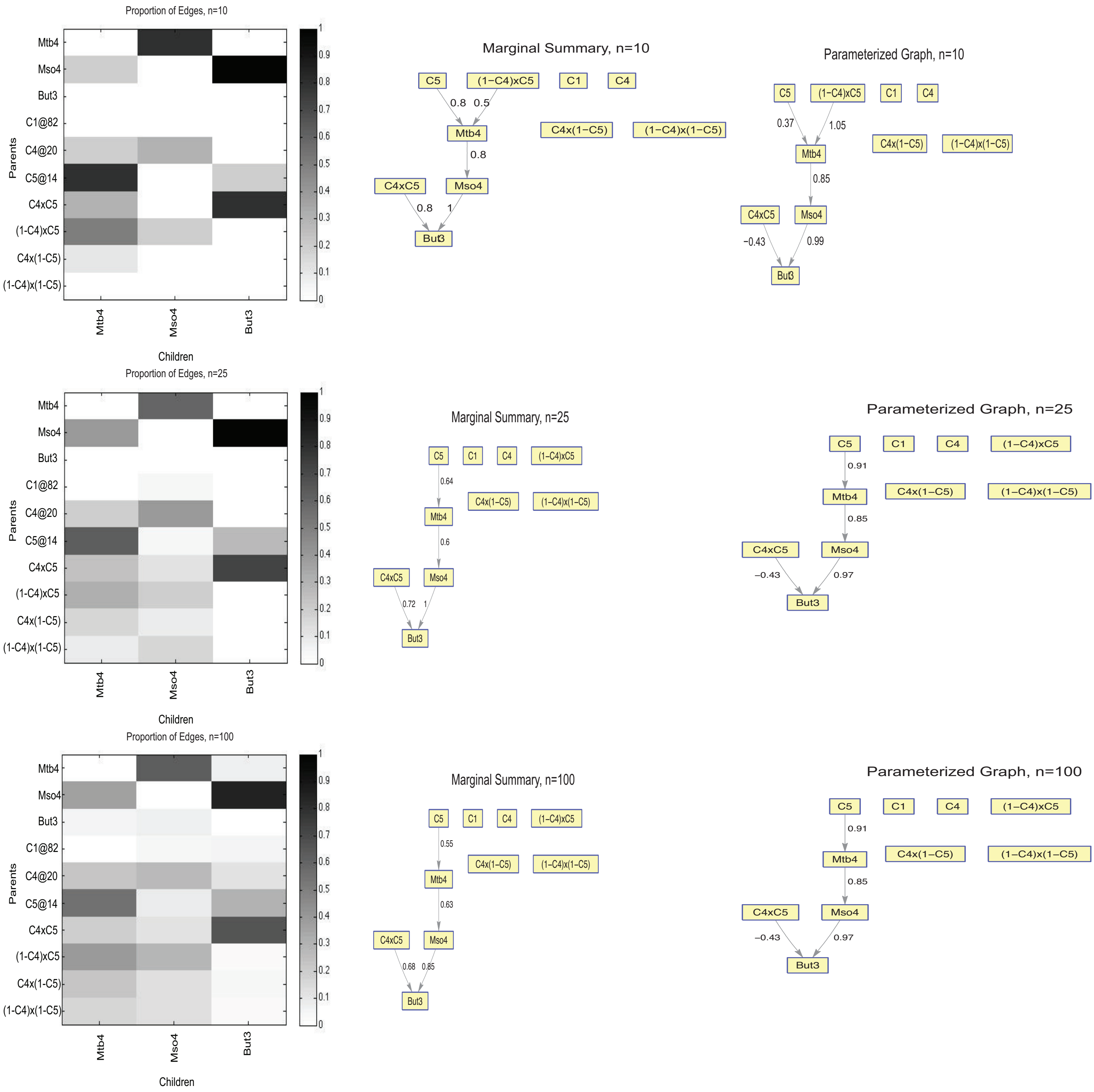

Figure 3. Dihomo-methionine sidechain network reconstructions. The results are shown for the top scoring n = 10, 25 and 100 graphs (top - bottom) identified through MCMC sampling. For each n the proportion of edges present in the summary, marginal summary based on edge proportion of at least 0.5 and the parameterized model is shown (left - right).

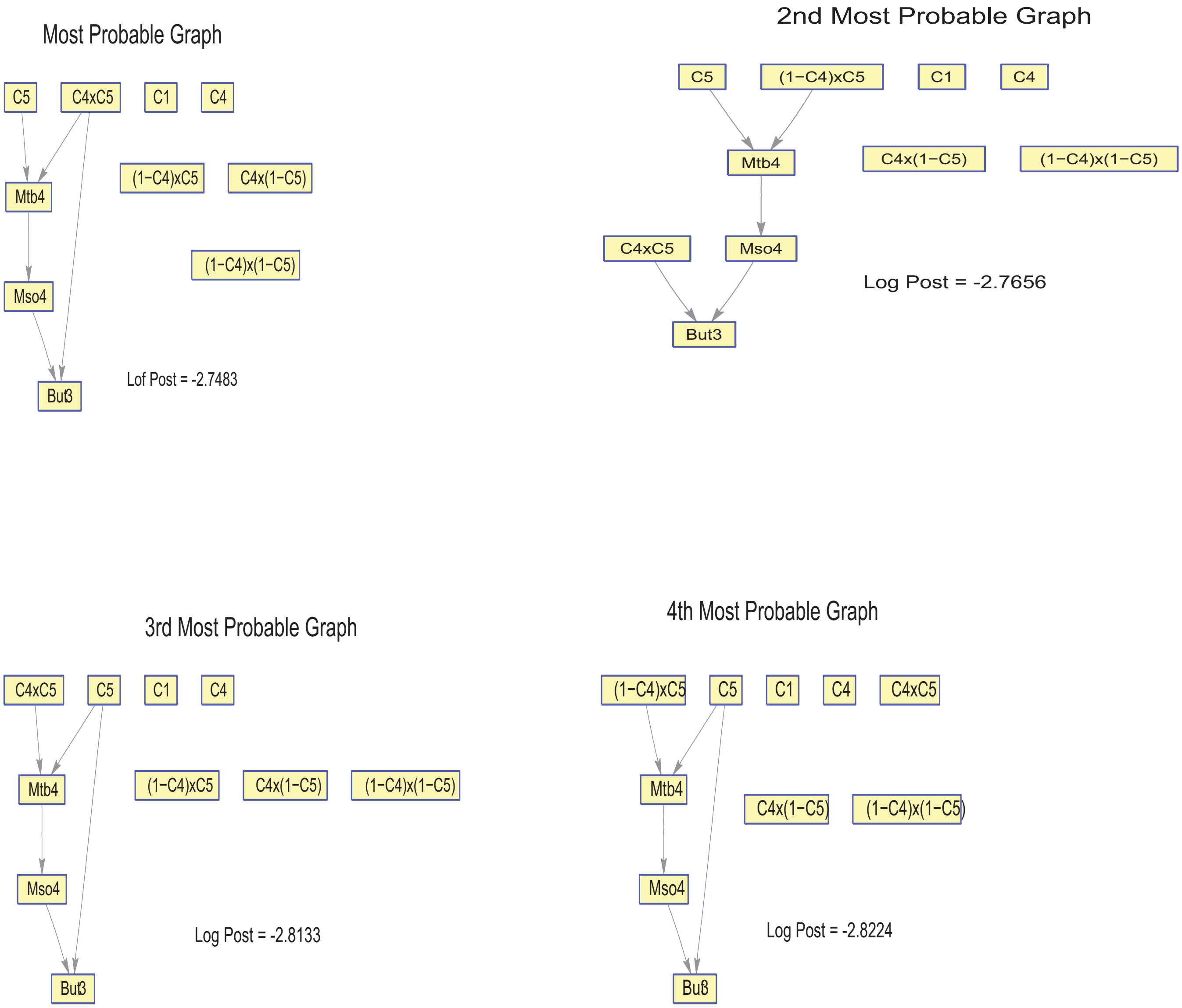

Figure 4. Dihomo-methionine sidechain top four parameterized network reconstructions and their posterior probabilities.

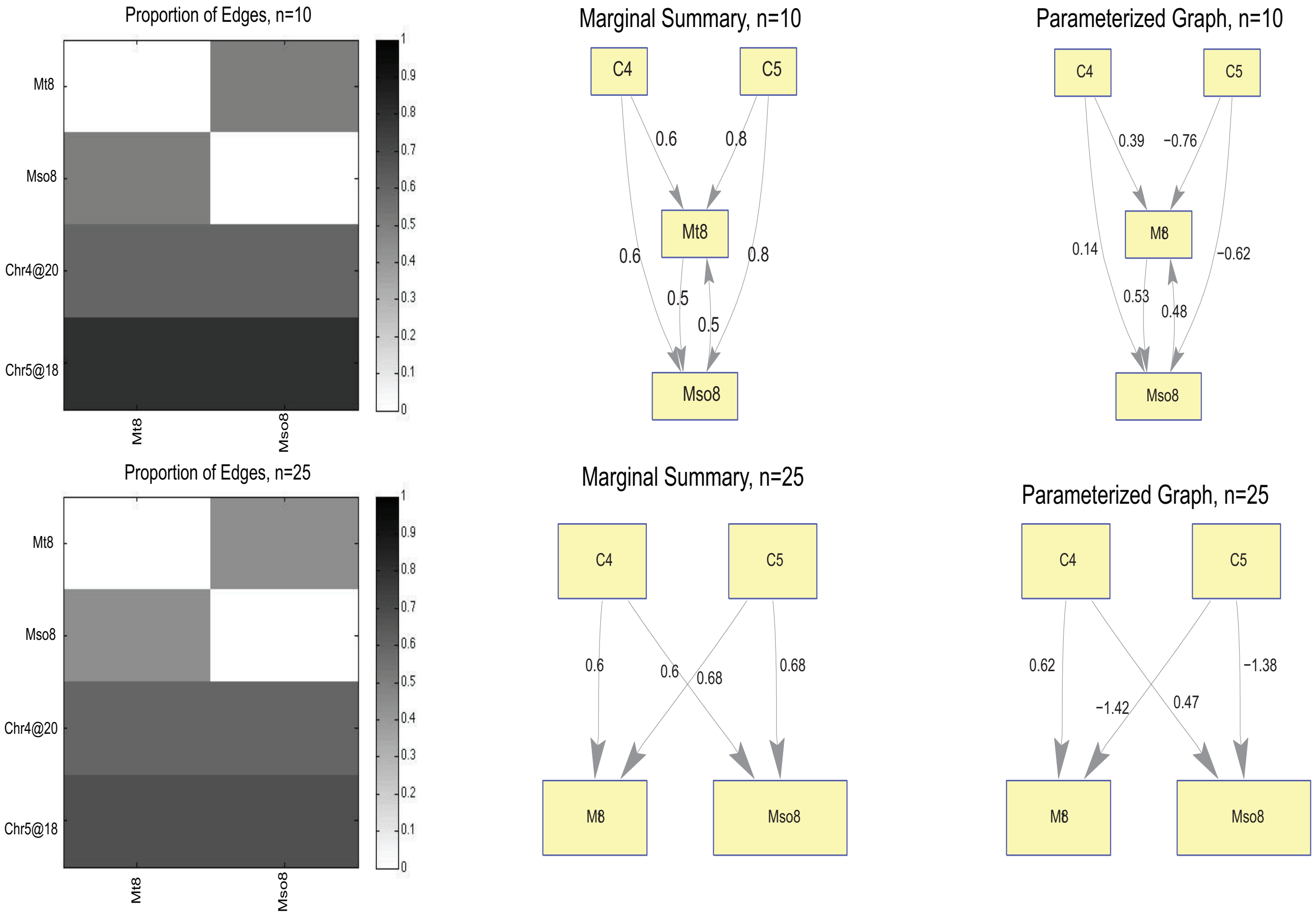

Figure 5. Hexa-methionine sidechain network reconstructions. The results are shown for the top scoring $n = 10$, 25 and 100 graphs (top - bottom) identified through MCMC sampling. For each n the proportion of edges present in the summary, marginal summary based on edge proportion of at least 0.5 and the parameterized model is shown (left - right).

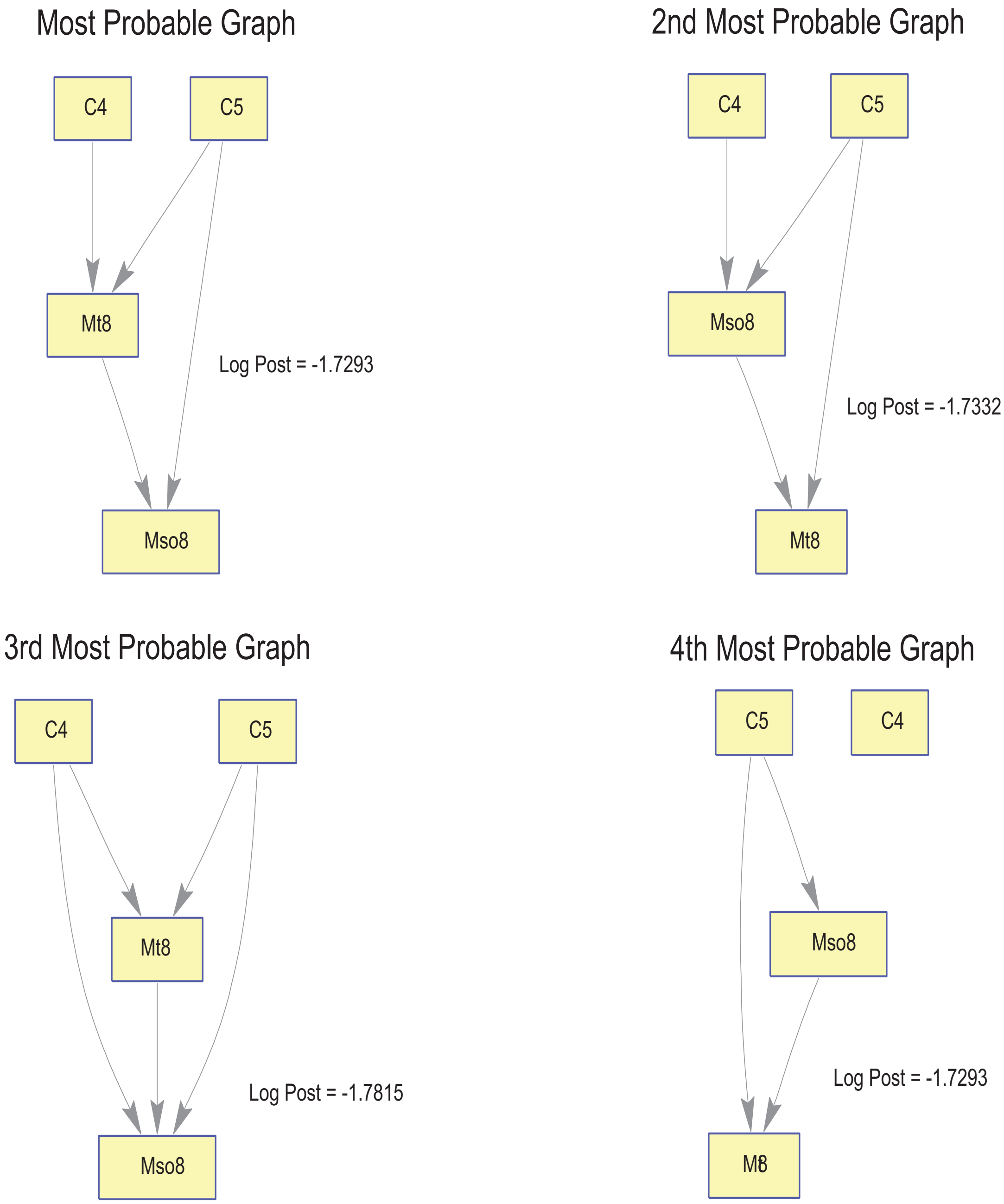

Figure 6. Hexa-methionine sidechain top four parameterized network reconstructions and their posterior probabilities.

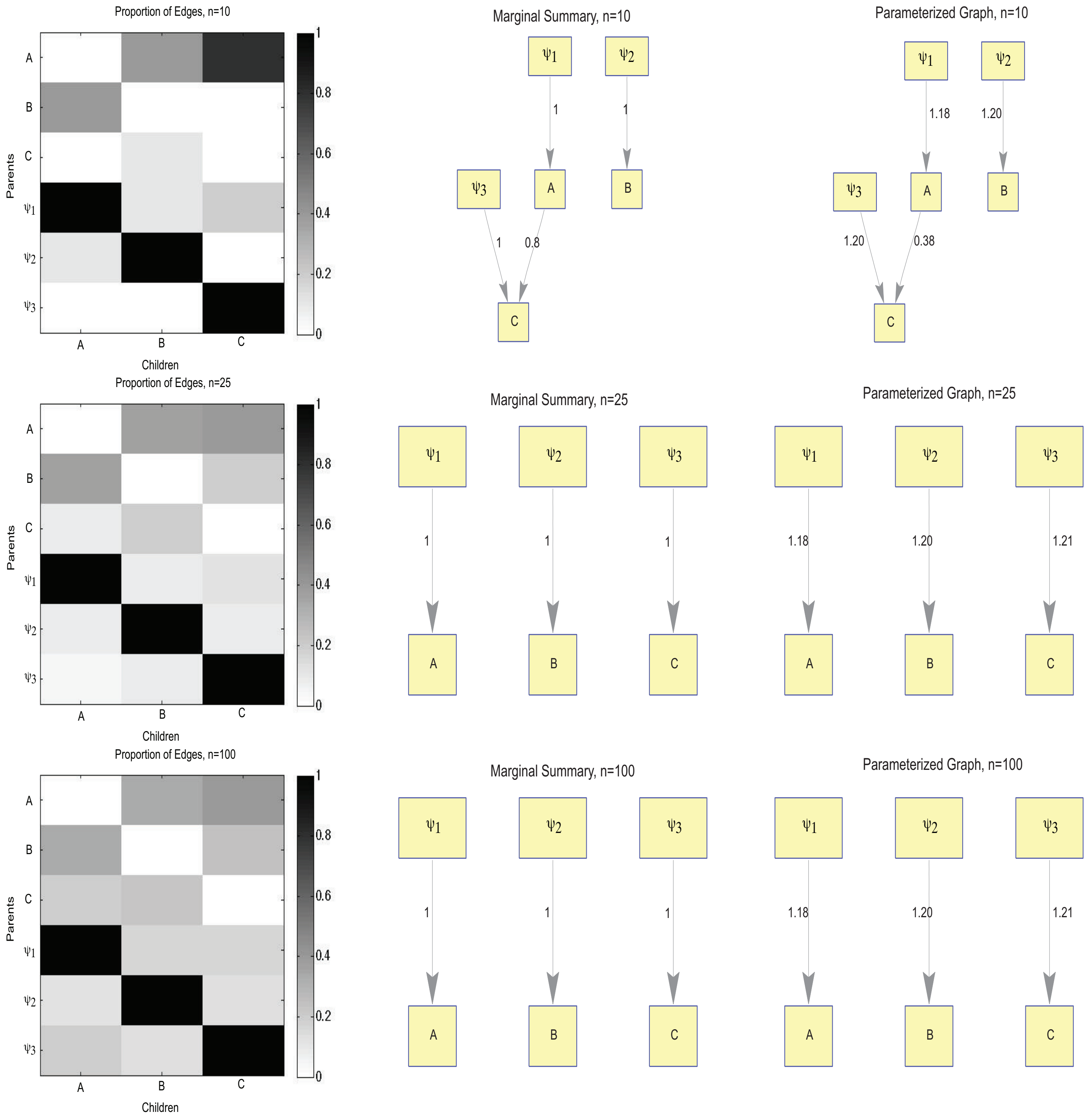

Figure 7. Linear pathway network reconstructions. The results are shown for the top scoring $n =$ 10, 25 and 100 graphs (top - bottom) identified through MCMC sampling. For each n the proportion of edges present in the summary, marginal summary based on edge proportion of at least 0.5 and the parameterized model is shown (left - right).

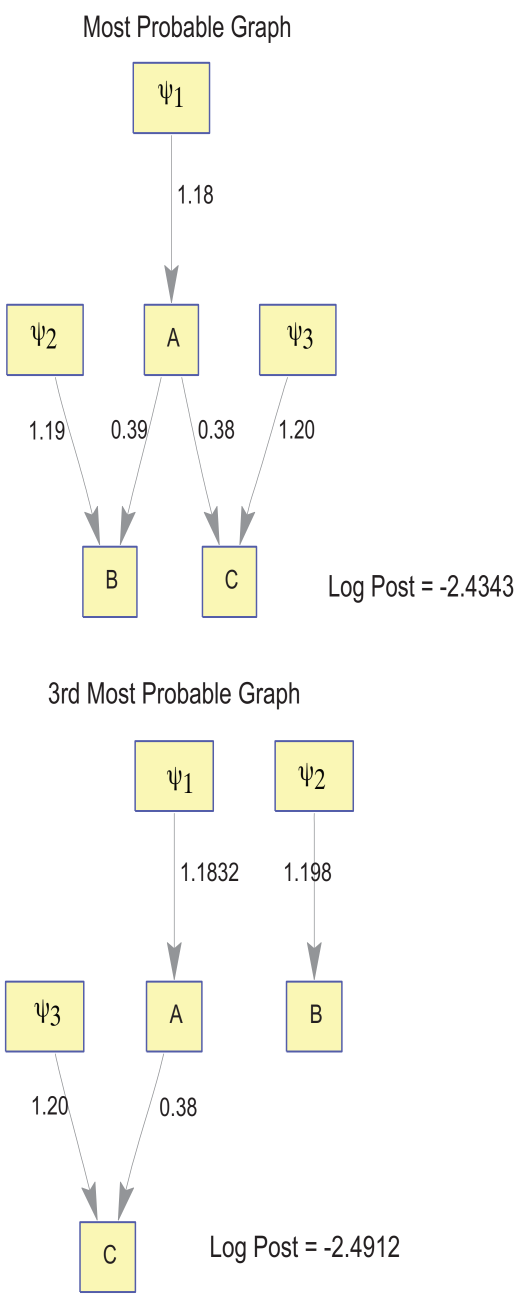

Figure 8. Linear pathway top four parameterized network reconstructions and their posterior probabilities.

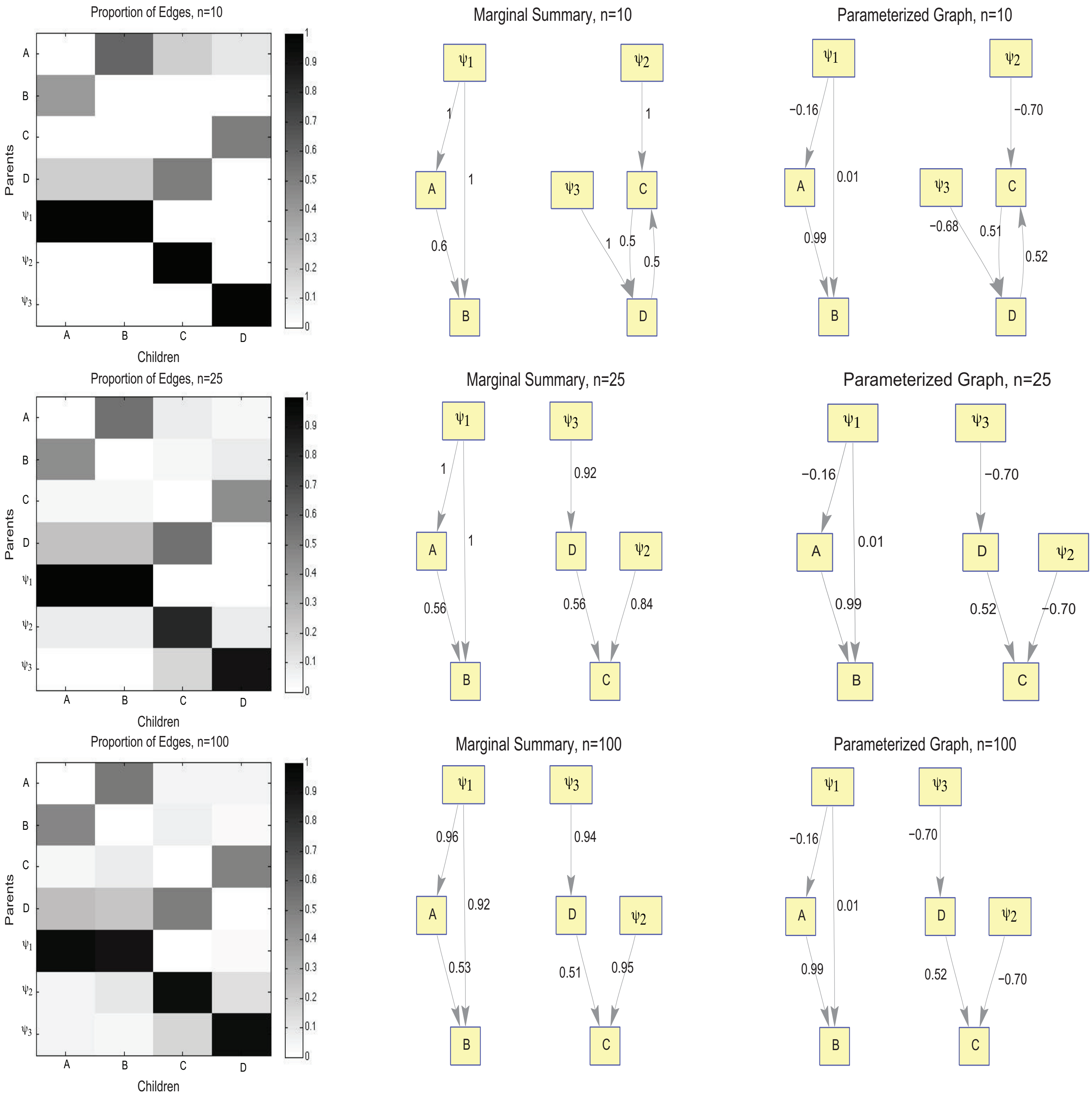

Figure 9. Merging pathway via metabolic reaction network reconstructions. The results are shown for the top scoring $n = 10$, 25 and 100 graphs (top - bottom) identified through MCMC sampling. For each n the proportion of edges present in the summary, marginal summary based on edge proportion of at least 0.5 and the parameterized model is shown (left - right).

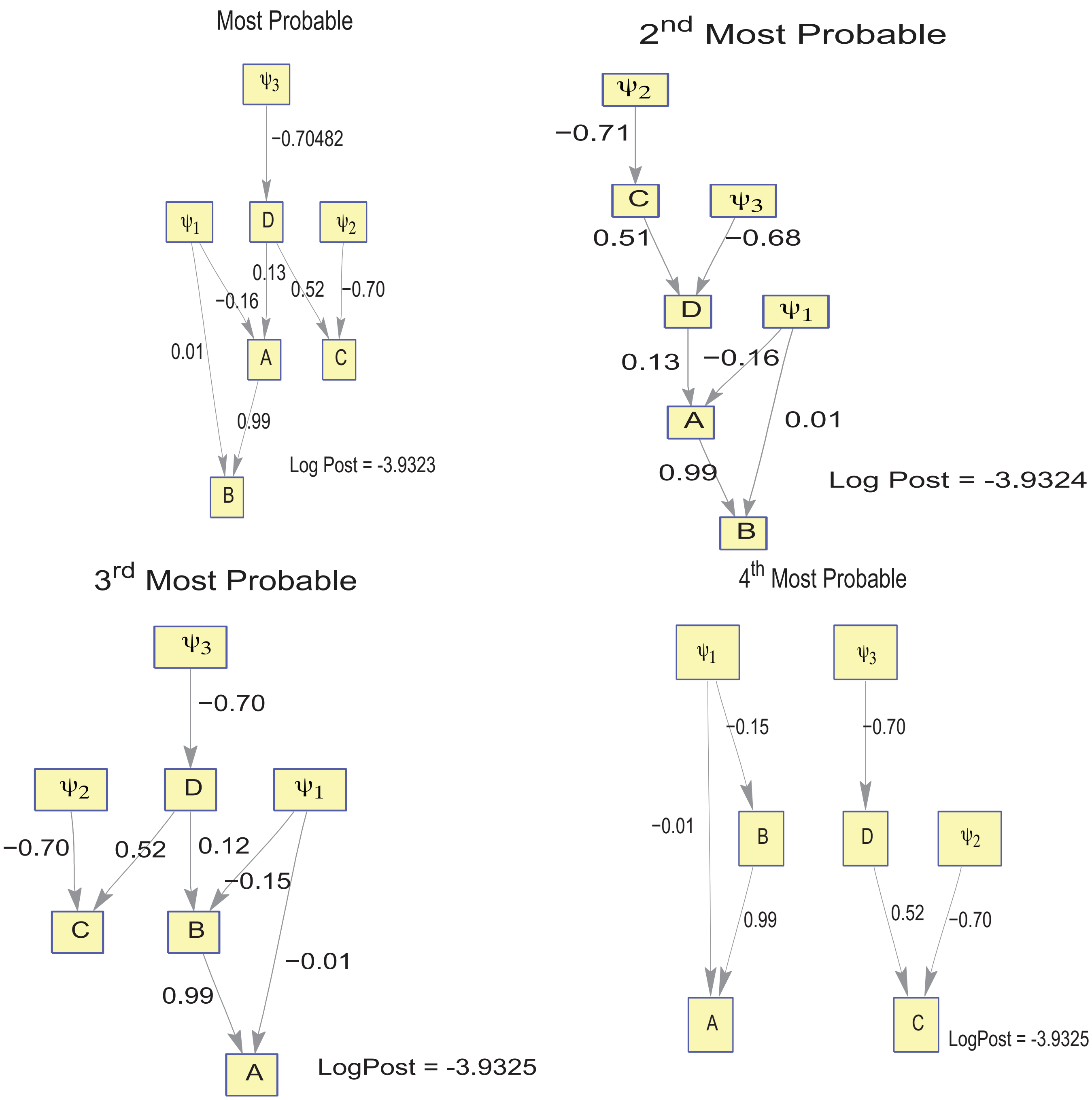

Figure 10. Merging pathway via metabolic reaction top four parameterized network reconstructions and their posterior probabilities.

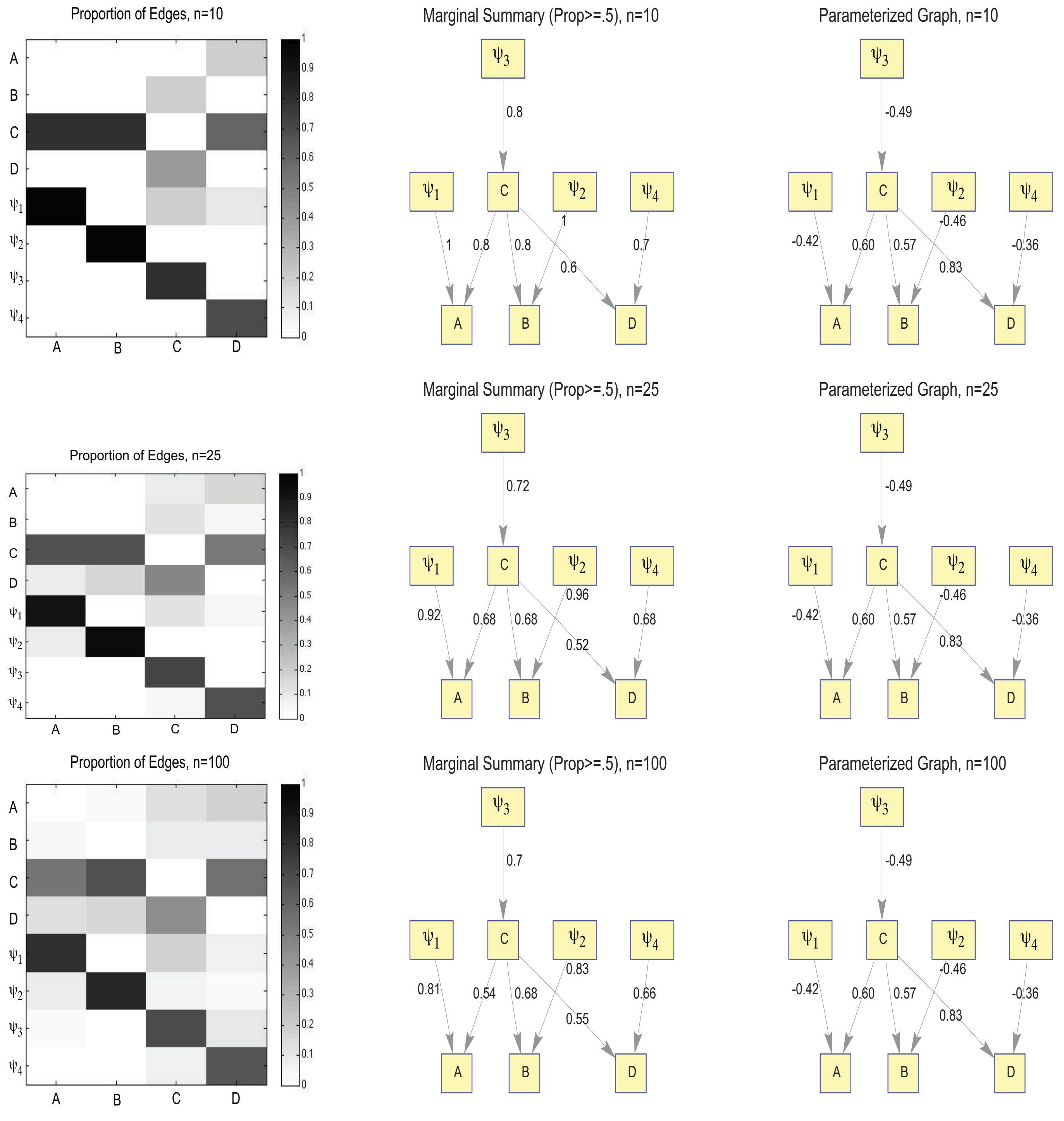

Figure 11. Merging pathway via independent paths network reconstructions. The results are shown for the top scoring $n = 10$, 25 and 100 graphs (top - bottom) identified through MCMC sampling. For each n the proportion of edges present in the summary, marginal summary based on edge proportion of at least 0.5 and the parameterized model is shown (left - right).

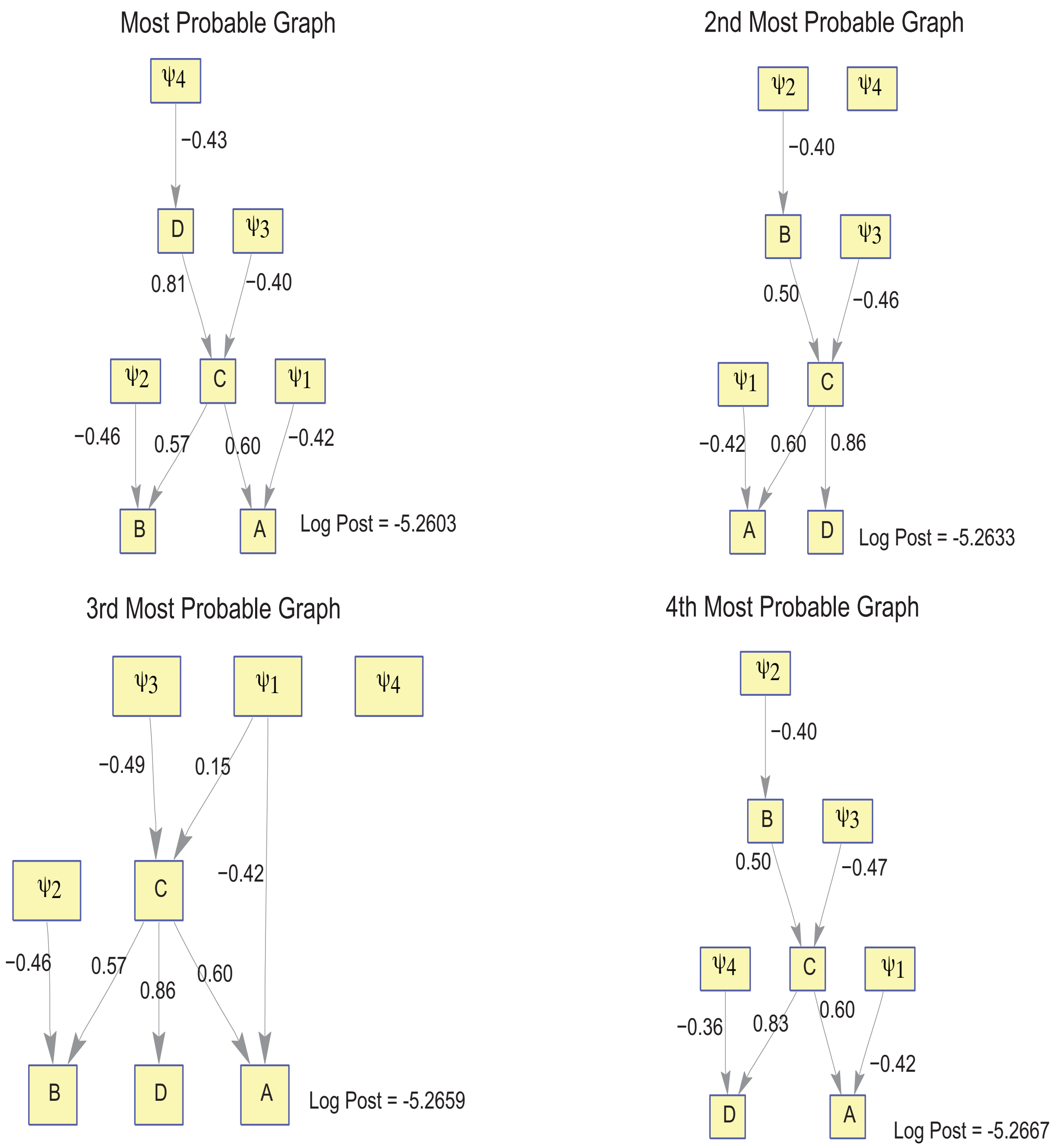

Figure 12. Merging pathway via independent paths top four parameterized network reconstructions and their posterior probabilities.

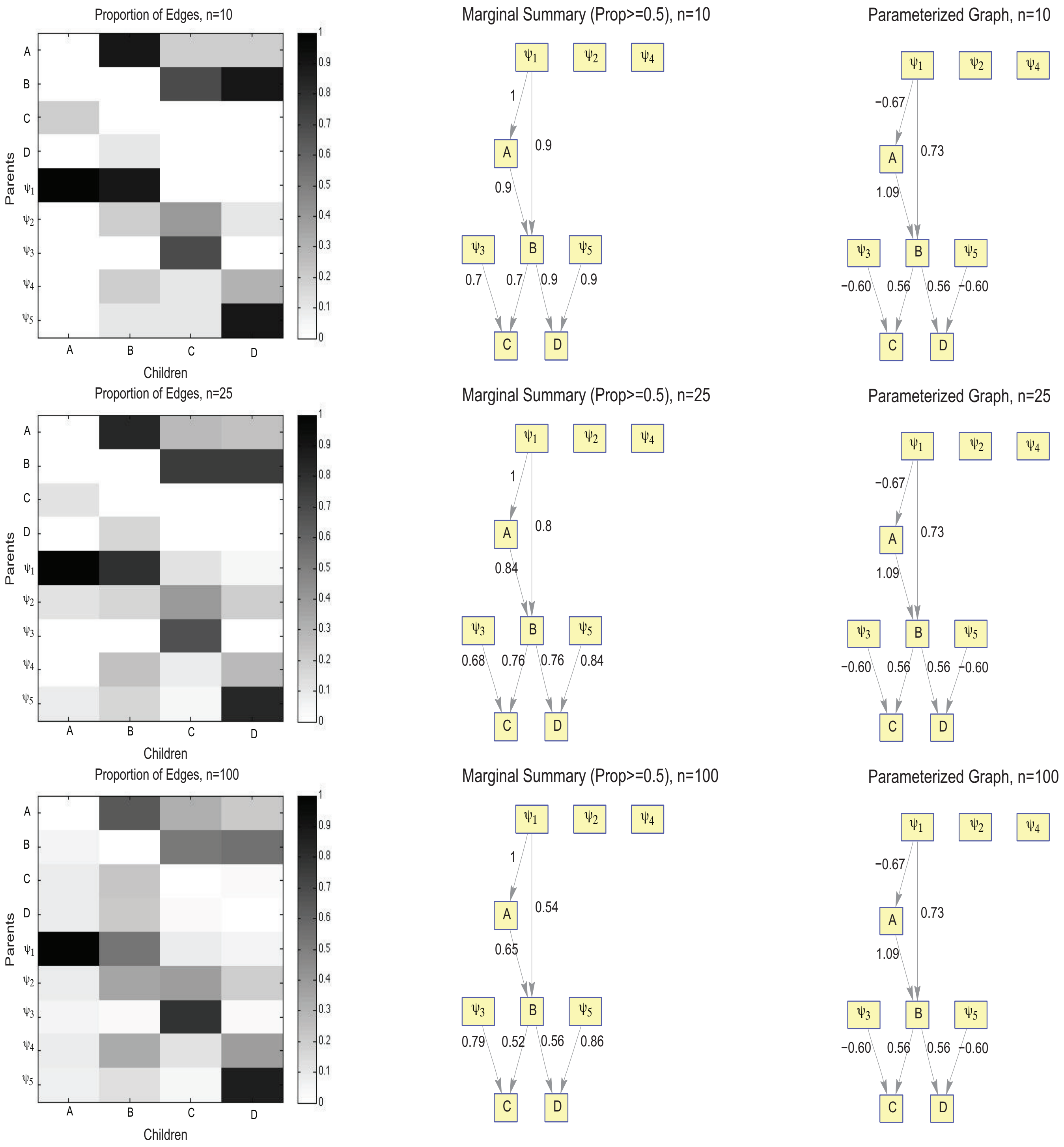

Figure 13. Branching pathway network reconstructions. The results are shown for the top scoring $n = 10$, 25 and 100 graphs (top - bottom) identified through MCMC sampling. For each n the proportion of edges present in the summary, marginal summary based on edge proportion of at least 0.5 and the parameterized model is shown (left - right).

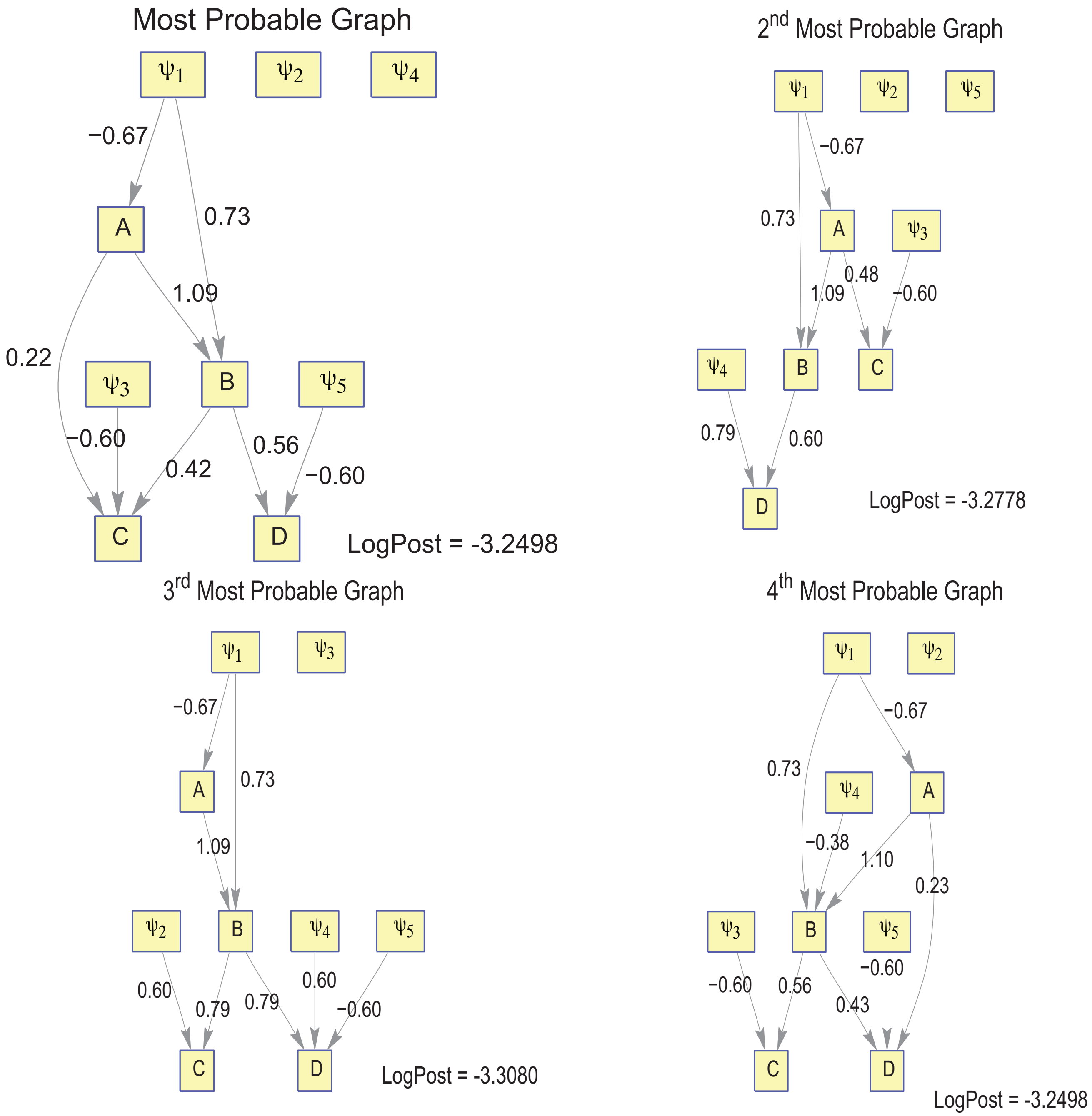

Figure 14. Branching pathway top four parameterized network reconstructions and their posterior probabilities.

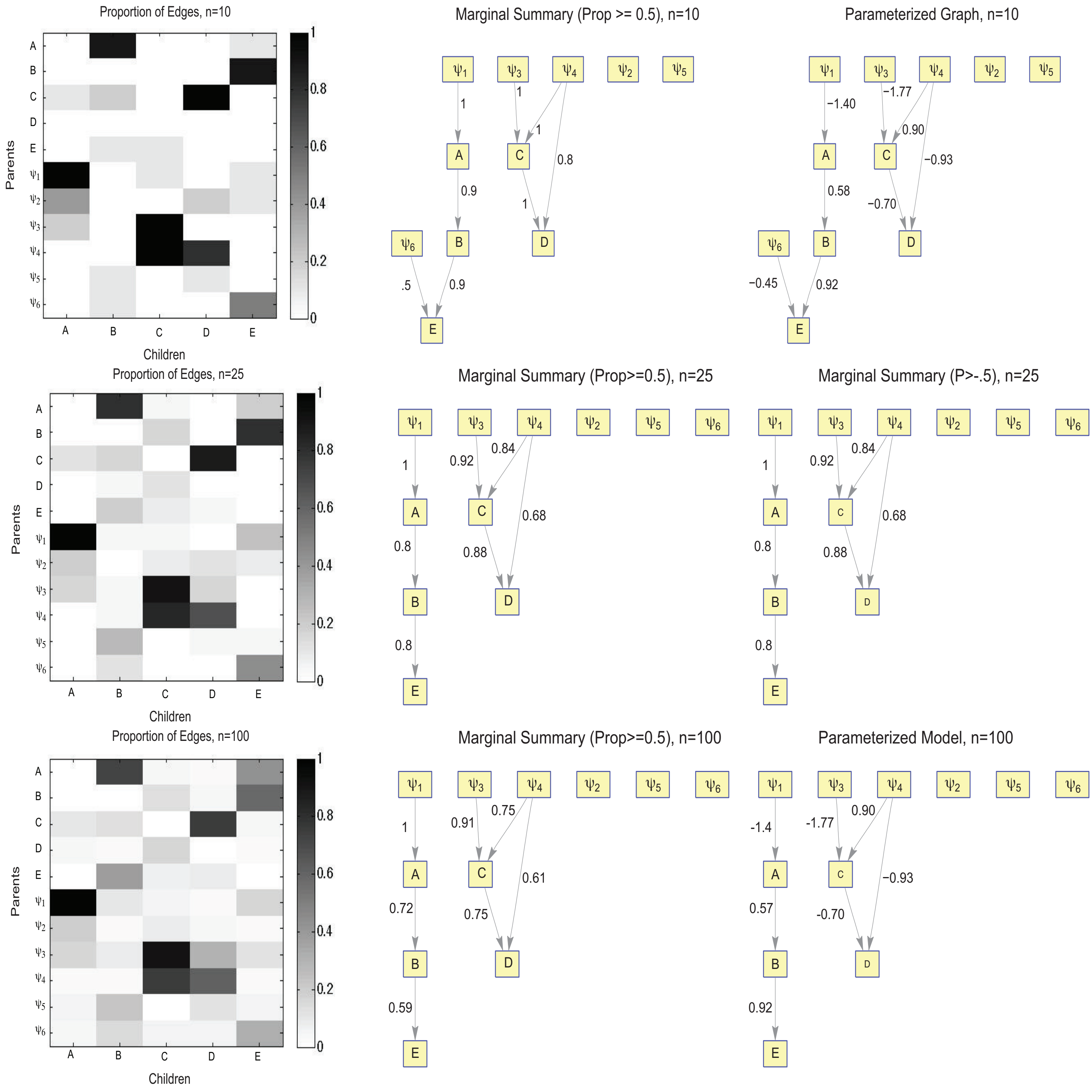

Figure 15. Branching pathway with metabolic inhibition network reconstructions. The results are shown for the top scoring n = 10, 25 and 100 graphs (top - bottom) identified through MCMC sampling. For each n the proportion of edges present in the summary, marginal summary based on edge proportion of at least 0.5 and the parameterized model is shown (left - right).

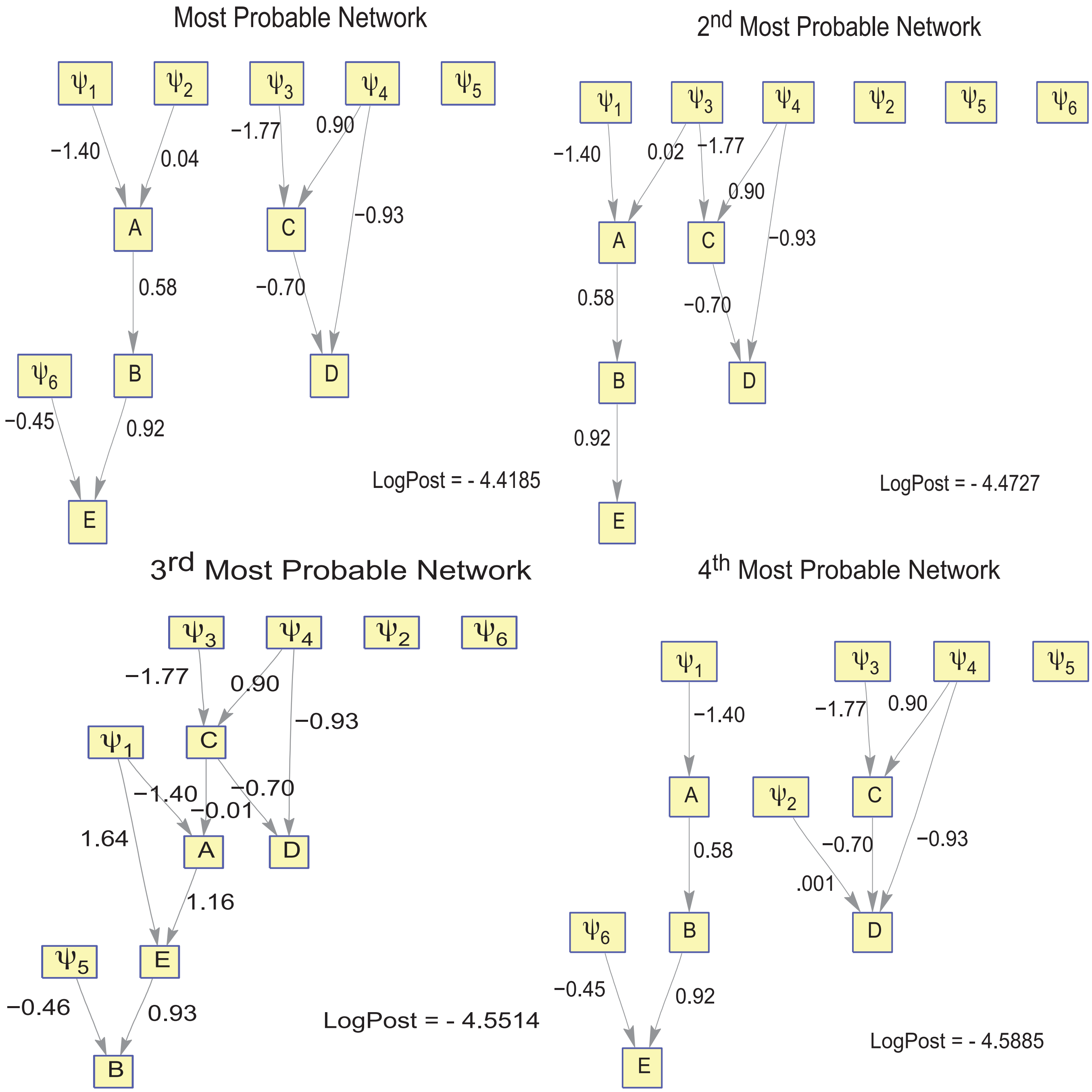

Figure 16. Branching pathway with metabolic inhibition top four parameterized network reconstructions and their posterior probabilities.
